# Dynamics of DNA Methylation and Its Functions in Plant Growth and Development

**DOI:** 10.3389/fpls.2021.596236

**Published:** 2021-05-21

**Authors:** Suresh Kumar, Trilochan Mohapatra

**Affiliations:** ^1^Division of Biochemistry, ICAR-Indian Agricultural Research Institute, New Delhi, India; ^2^Indian Council of Agricultural Research, New Delhi, India

**Keywords:** DNA methylation, DNA modification, environmental stress, epigenetics, gene regulation, 5-methylcytosine, N^6^-methyladenine, plant growth

## Abstract

Epigenetic modifications in DNA bases and histone proteins play important roles in the regulation of gene expression and genome stability. Chemical modification of DNA base (e.g., addition of a methyl group at the fifth carbon of cytosine residue) switches on/off the gene expression during developmental process and environmental stresses. The dynamics of DNA base methylation depends mainly on the activities of the writer/eraser guided by non-coding RNA (ncRNA) and regulated by the developmental/environmental cues. *De novo* DNA methylation and active demethylation activities control the methylation level and regulate the gene expression. Identification of ncRNA involved in *de novo* DNA methylation, increased DNA methylation proteins guiding DNA demethylase, and methylation monitoring sequence that helps maintaining a balance between DNA methylation and demethylation is the recent developments that may resolve some of the enigmas. Such discoveries provide a better understanding of the dynamics/functions of DNA base methylation and epigenetic regulation of growth, development, and stress tolerance in crop plants. Identification of epigenetic pathways in animals, their existence/orthologs in plants, and functional validation might improve future strategies for epigenome editing toward climate-resilient, sustainable agriculture in this era of global climate change. The present review discusses the dynamics of DNA methylation (cytosine/adenine) in plants, its functions in regulating gene expression under abiotic/biotic stresses, developmental processes, and genome stability.

## Introduction

Methylation of DNA bases at different positions (e.g., fifth carbon of cytosine and N^6^ of adenine) plays significant roles in epigenetic regulation of gene expression in both plants and animals (Zhang et al., 2006; Xiang et al., 2010; Kumar et al., 2018). Epigenomic changes such as methylation of DNA bases, modification of histone proteins, and changes in the biogenesis of non-coding RNAs (ncRNAs) influence chromatin structure (accessibility of the genetic information to transcriptional machinery), thus gene expression, and genome integrity/stability. Methylation of DNA bases is known to be an important regulator of biological processes, and interruption in DNA methylation homeostasis leads to several developmental abnormalities in plants (e.g., *Arabidopsis thaliana*) and animals (e.g., mice; Slotkin and Martienssen, 2007; Lang et al., 2017). While DNA methylation is catalyzed by different methyltransferases (using S-adenosyl-l-methionine as a methyl group donor), active DNA demethylation uses enzyme-catalyzed base excision repair (BER) pathway (Penterman et al., 2007; Kumar et al., 2018; Li et al., 2018a). Although the RNA-directed DNA methylation (RdDM) pathway is vital for *de novo* DNA methylation in plants, it is not so important in mammals (Matzke and Mosher, 2014). Active DNA demethylation initiates with deamination and/or oxidation of 5-methylcytosine (5-mC) in mammals, but in plants, direct excision of 5-mC takes place using methylcytosine DNA glycosylase (Law and Jacobsen, 2010; Li et al., 2018a). Besides, covalent but reversible posttranslational histone modifications and interaction with DNA play important role in regulating chromatin condensation and DNA accessibility (Ooi et al., 2006; Wei et al., 2017). Various mechanisms involved in site-specific DNA base modifications and their functions in the regulation of gene expression are being deciphered in model plants like *Arabidopsis* (Wang et al., 2016; Pecinka et al., 2019). N^6^-methyladenine (6-mA) is another important modified DNA base (comparatively less abundant in plants) playing regulatory functions in animals and plants. It is considered to be essential for growth and development in *Arabidopsis* and rice (Liang et al., 2018; Xiao et al., 2018; Zhang et al., 2018b). Generally, a mutation in the gene encoding for component of DNA (de)methylation machinery or a regulatory factor does not cause lethality of the individual. Though *Arabidopsis* has been used as a model plant to understand the basic epigenetic machinery, the gathered information is validated and variations are being mapped in crop plants like rice (*Oryza sativa* L.). Efforts are also being made to identify the epigenetic marks associated with a trait of interest so that they can be utilized in crop improvement programs toward the development of climate-smart crops (Varotto et al., 2020). Nevertheless, DNA modifications appear to be crucial for developmental processes and protection from environmental stresses. Recent findings are unraveling the components (readers, writers, erasers, etc.) involved in DNA modification in plants. Such a recent understanding includes the necessity of a methylation-sensing genetic element in maintaining DNA (de)methylation homeostasis (Lei et al., 2015; Williams et al., 2015), the contribution of ncRNA in triggering *de novo* DNA methylation (Ye et al., 2016), and the role of increased DNA methylation protein in targeted DNA demethylation (Duan et al., 2017). The present review discusses the dynamics of DNA base methylation and its functions, particularly in controlling the activity of transposable elements (TEs), genome stability, regulation of gene expression during plant growth, development, and environmental stress.

## Dynamics of DNA Methylation

Variation in DNA methylation has been detected in many organisms, including viruses, prokaryotes, and eukaryotes (Berdis et al., 1998; Bell and Felsenfeld, 2000; Hoelzer et al., 2008). Methylation of DNA plays important roles in the regulation of gene expression, growth, development, and protection from environmental stresses, as well as in stabilizing the genome (Zilberman et al., 2006; Mendizabal and Yi, 2016; Kumar et al., 2017a, 2018). DNA base modification in a context-and genomic region-specific manner is catalyzed by different enzymes through distinct pathways. Methylcytosine (5-mC), also known as the fifth base of DNA, was discovered long before the DNA was recognized as genetic material in a living cell. Although more attention is given to the conventional 5-mC, recent findings on additional base modifications [e.g., hydroxymethylcytosine (5-hmC), formylcytosine (5-fC), carboxylcytosine (5-caC), and N^6^-methyladenine (6-mA)] have resulted in overwhelming interest in epigenomic studies. In plants, cytosine methylation can occur in all contexts of cytosine (CG, CHG, and CHH, where H=A, C, or T; Lister et al., 2008; Wang et al., 2016). In *Arabidopsis* as well as in other plants, the heterochromatic regions are enriched with methylcytosines, generally in the repetitive sequences and TEs. However, TEs and 5-mC are also found to be interspersed in the euchromatic regions (Zhang et al., 2006; Rathore et al., 2020). The dynamics of DNA base methylation depends on the reversibility of the processes, which also controls switching on/off the gene. Diversity and complexity of epigenetic changes (DNA/histone modifications and ncRNA biogenesis) in different organisms are being discovered continuously, and the potential combinatorial interactions of epimarks indicate that epigenetic codons would be considerably more complex than it is thought today (Kumar et al., 2018).

### Cytosine Methylation

Establishment, maintenance, and removal of cytosine methylation in different contexts/genomic regions in the plant genome occur through various pathways. While *de novo* cytosine methylation involves the RdDM pathway, maintenance of cytosine methylation in different sequence contexts depends on various DNA methyltransferases. Removal of 5-mC might occur either due to the malfunction of methyltransferase, scarcity of methyl donor (S-adenosylmethionine, AdoMet) during passive DNA demethylation, or by the active DNA demethylation process. In active DNA demethylation, a family of enzymes [bifunctional 5-methylcytosine DNA glycosylases–apurinic/apyrimidinic lyase (APE1L)] initiate the demethylation process *via* BER pathway (Almeida and Sobol, 2007; Li et al., 2018a). While promoter methylation is generally associated with switching-off/downregulation of the gene, methylation of the coding sequence may have negative or positive effects on gene expression (Takuno and Gaut, 2013; Williams et al., 2015; Kumar et al., 2017a).

**Figure 1:**
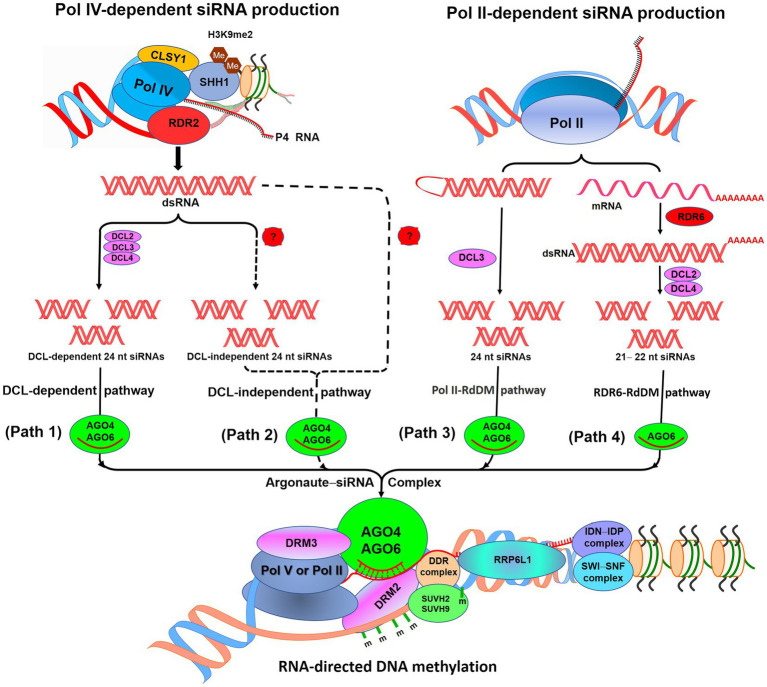
Diagrammatic representation of the RNA-directed DNA methylation (RdDM) pathway. According to the canonical RdDM pathway, noncoding P4 RNAs are produced by RNA polymerase IV (Pol IV). SHH1 binds to dimethylated histone H3K9me2 and helps to recruit Pol IV at RdDM locus. (Path 1): P4 RNAs get converted into double-stranded RNAs (dsRNAs) by RDR2, which get cleaved into 24 nucleotide (nt) siRNAs by DICER-like protein 2 (DCL2),ss DCL3, and DCL4. These siRNAs bound with Argonaute 4 or AGO6 participate in the RdDM. (Path 2): Methylation of RdDM loci in *dcl1*-*dcl2*-*dcl3*-*dcl4* mutant suggests the existence of DCL-independent RdDM. (Path 3): POL II produces 24 nt siRNAs with the help of DCL3 and scaffold RNAs at some of the RdDM loci. (Path 4): For some active transposons, mRNAs get converted into dsRNAs and get cleaved into 21 nt siRNAs by DCL2, DCL4 through RDR6–RdDM pathway. Involved in *de novo* (IDN)–IDN2 Paralog (IDP) complex and RNA-binding proteins RRP6-like 1 (RRP6L1) interact with a chromatin-remodeling complex Switch/Sucrose Nonfermenting (SWI/SNF) to facilitate retention of nascent Pol V-transcribed RNA. m, methylcytosine. (Redrawn from Zhang et al., 2018a).

RdDM pathway is responsible for *de novo* methylation of DNA which utilizes small-interfering RNAs (siRNAs), scaffold RNAs, and many accessory proteins (Figure 1). Present understanding of the RdDM pathway in *Arabidopsis* (Law and Jacobsen, 2010; Matzke and Mosher, 2014; Zhang et al., 2018a) suggests that RNA polymerase IV (Pol IV) initiates the production of 24 nt siRNA (noncoding P4 RNA) which serves as the template for RNA-dependent RNA polymerase 2-mediated generation of double-stranded RNAs (dsRNA). Sawadee Homeodomain Homolog 1 helps in the recruitment of Pol IV to the RdDM-targeted loci having dimethylated histone H3 lysine 9 (H3K9me2; Law et al., 2013; Zhang et al., 2013a). An SNF2 domain-containing protein Classy 1 (CLSY1), a chromatin remodeler, interacts with Pol IV, which is necessary for Pol IV-dependent siRNA production (Zhang et al., 2013a). DICER-like protein 2 (DCL2), DCL3, and DCL4 cleave the dsRNAs to generate 24 nt siRNAs (DCL-dependent siRNA production). Many of the RdDM-targeted loci were reported to remain methylated in quadruple (*dcl1*-*dcl2*-*dcl3*-*dcl4*) mutant; this suggests that siRNAs may also be produced by DCL-independent RdDM pathway or directly from P4 RNAs (Yang et al., 2016). At some of the RdDM-targeted loci, Pol II-dependent siRNA production starts with the production of 21–24 nt siRNAs. While transcription of some of the intergenic loci by Pol II produces 24 nt siRNAs and scaffold RNAs, transcription of some activated transposons by Pol II and RNA-dependent RNA polymerase 6 (RDR6) produces 21 or 22 nt siRNA precursors in association with DCL2 and DCL4 (Wu et al., 2012; Nuthikattu et al., 2013; McCue et al., 2015).

Subsequently, siRNA gets loaded onto Argonaute (AGO) proteins (AGO4 and/or AGO6) and directly associated with Pol V-transcribed scaffold RNAs which finally recruit domains rearranged methylase 2 (DRM2, a DNA methyltransferase) for methylation of the target locus. Interaction of AGO4 with DRM2 catalyzes *de novo* methylation of cytosine in a sequence-independent manner (Zhong et al., 2014). AGO association with Pol IV is complemented by RNA-directed DNA methylation 3 (Bies-Etheve et al., 2009). Generation of the scaffold RNAs requires DDR complex (consisted of a chromatin remodeler defective in RNA-directed DNA methylation 1, and defective in meristem silencing 3), which also associates with AGO4/AGO6, single-stranded methylated DNA, and DRM2 (Gao et al., 2010; Law et al., 2010; Zhong et al., 2012; Liu et al., 2014). The DDR complex also interacts with the suppressor of variegation 3-9 homolog protein 2 (SUVH2) and SUVH9 which bind together to the preexisting methylcytosine and help recruiting Pol V (Zhong et al., 2012; Johnson et al., 2014). SUVH2 and SUVH9 recognize methylcytosine through their RING finger-associated and SET domains which are needed for genome-wide chromatin binding of Pol V through preexisting DNA methylation. The binding of SUVH9, also having zinc finger, even to the unmethylated DNA was reported to be sufficient enough to recruit Pol V for methylation of DNA and silencing of the gene (Johnson et al., 2014). Pol V can produce ncRNAs with different 5′ ends from a locus, which indicates that it can start transcription without a promoter (Wierzbicki et al., 2008). The Pol V-generated scaffold RNAs are long enough to be detected by PCR and lack polyadenylation at 3′ end; thus, they differ from mRNA (Wierzbicki et al., 2008).

**Figure 2:**
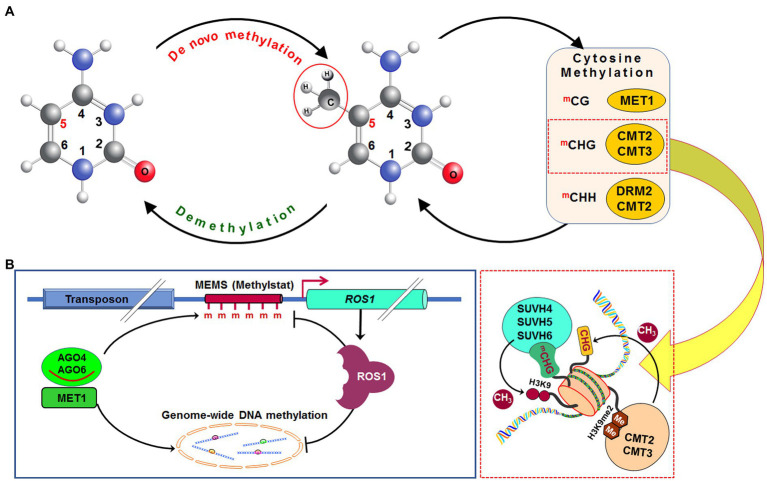
Dynamics of DNA methylation in plants. *De novo* DNA methylation occurs in all (CG, CHG, and CHH; where H=A, C, or T) cytosine contexts. After replication of DNA, methylation in the CG context is maintained by methyltransferase 1 (MET1), while methylation in CHG context is maintained by chromomethylase 2 (CMT2) or CMT3, and methylation in CHH context is maintained by CMT2 or by DRM2 *via* RdDM pathway. Methylated CHG (^m^CHG) attracts histone H3 lysine 9 (H3K9)-specific suppressor of variegation 3-9 homolog protein 4 (SUVH4), SUVH5, and SUVH6 and generates dimethylated H3K9 (H3K9me2), which enables CMT2 and CMT3 **(A)**. Methylation of methylation monitoring sequence (MEMS), also known as “methylstat” present in the promoter of the Repressor of silencing 1 (*Ros1*) is necessary for transcription of the *Ros1* gene. Cytosine methylation at MEMS is controlled by MET1/RdDM and Ros1 itself. This helps to sense/monitor the level of methylation and regulate DNA (de)methylation homeostasis **(B)**. CH_3_, methyl group, Me/m, methylation (Redrawn from Zhang et al., 2018a).

Methylation of cytosine in hemimethylated CG dinucleotide, created due to DNA replication, is performed by methyltransferase 1 (MET1), an orthologue of DNA methyltransferase 1 in mammals. It adds methyl (CH_3_) group at fifth carbon of cytosine in daughter strand of the replicated DNA (Figure 2A). Recruitment of MET1 to the hemimethylated CG is mediated by variant in methylation proteins, which are UHRF1 orthologs (Woo et al., 2008). Methylation at CHG context in the daughter DNA strand is catalyzed mainly by chromomethylase 3 (CMT3) and to some extent by CMT2 (Stroud et al., 2014). SUVH4, SUVH5, and SUVH6 bind to the methylated CHG domain and facilitate the CMT3/CMT2 function (Du et al., 2012, 2014; Stroud et al., 2013). Mutation in SUVH4, SUVH5, and SUVH6 was reported to reduce CHG methylation in *Arabidopsis* (Ebbs and Bender, 2006; Stroud et al., 2013). Moreover, methylation at asymmetric CHH context is performed by DRM2 or CMT2 depending on the nature of the genomic region. At shorter transposons and repeat sequences in euchromatic regions, as well as at longer transposons in heterochromatin, DRM2 causes CHH methylation through the RdDM pathway (Zemach et al., 2013; Liu et al., 2014). Mutation in decreased DNA methylation 1 (DDM1), a chromatin-remodeling protein, causes impaired methylation by CMT2; DRM2 and CMT2 can also methylate cytosine in other contexts (Zhang et al., 2018a).

### Demethylation of 5-Methylcytosine

Replacing 5-mC with cytosine (unmethylated) is an equally important phenomenon in the regulation of gene expression through DNA methylation. Thus, methylation level is dynamically maintained by DNA (de)methylation. Passive (non-enzymatic) DNA demethylation occurs due to the loss of methylase activity during DNA replication (Mayer et al., 2000; Li et al., 2018a). Passive demethylation (reduced expression of MET1) was proposed to be responsible for demethylation in the central cell of female gametophyte, which develops into endosperm in seed after fertilization (Jullien et al., 2008; Kawashima and Berger, 2014). However, Park et al. (2016) recently reported maintenance of the methylation level in the central cell of *Arabidopsis* and rice. Calarco et al. (2012) reported maintenance of methylation in CG and CHG contexts during microsporogenesis which might be responsible for epigenetic inheritance in *Arabidopsis*. The vegetative nucleus in pollen shows very high methylation in CHH context, while the sperm cells show reduced CHH methylation due to reduced RdDM activity.

DNA methylation is also erased by active (enzymatic) DNA demethylation. While the active DNA demethylation process requires a family of enzymes, only one enzyme (methyltransferase) can accomplish the methylation process. In mammals, active DNA demethylation occurs through the BER pathway deploying DNA glycosylase wherein a 5-mC gets removed by TET dioxygenase-mediated oxidation of 5-hmC (Wu and Zhang, 2017). But in plants, a family of bifunctional DNA glycosylases–APE1Ls initiates the process through the BER pathway (Li et al., 2018a). Plant DNA glycosylase binds to 5-mC and removes it directly by breaking the glycosylic bond between the base and deoxyribose sugar. Subsequently, it acts as APE1Ls and breaks the DNA backbone producing an abasic site. APE1L and ZDP (a DNA polynucleotide 3′-phosphatase) generate 3′ OH; later on, the gap gets filled by the actions of DNA polymerase and ligase (Martinez-Macias et al., 2012; Lee et al., 2014; Li et al., 2015). In *Arabidopsis*, four known bifunctional DNA glycosylases include Repressor of silencing 1 (Ros1), Demeter (DME), Demeter-like protein 2 (DML2), and DML3 (Ortega-Galisteo et al., 2008). These glycosylases can remove 5-mC from any sequence context (Morales-Ruiz et al., 2006; Penterman et al., 2007; Zhu et al., 2007). DME is preferentially expressed in the companion (vegetative) cell of male and central cell of female gametes (Huh et al., 2008).

DME-favored demethylation of AT-rich TEs in euchromatin leads to changes in the expression of the nearby genes (Gehring et al., 2009; Hsieh et al., 2009; Ibarra et al., 2012). ROS1 demethylates TEs, which affects transposon activity and transcriptional silencing of the nearby gene (Tang et al., 2016). ROS1 also demethylates the RdDM-independent regions (He et al., 2009; Gao et al., 2010). The genomic regions targeted for ROS1-mediated demethylation are characterized by reduced H3K27me and/or H3K9me2, and enhanced H3K18Ac and/or H3K27me3 epimarks (Tang et al., 2016). At certain ROS1 target cites, chromatin environment legitimate for ROS1 active DNA demethylation is founded by the binding of histone acetyltransferase increased DNA methylation 1 at methylated DNA, which acetylates H3 particularly at the sites deprived of H3K4me2 and H3K4me3 (Qian et al., 2012).

The promoter of *ROS1* contains a 39 bp cytosine methylation monitoring sequence (*MEMS*), which has decreased methylation *in met1* and *RdDM* mutants (Figure 2B). Since hypomethylation of *MEMS* is accompanied by repression *of ROS1*, it indicates *MEMS* to function as a sensor/indicator of RdDM and MET1 activities. Thus, *MEMS* coordinates the methylation and demethylation processes through *ROS1* expression (Lei et al., 2015). *ROS1* promoter also contains a Helitron transposon upstream of the *MEMS*, which attracts cytosine methylation factors, and thus makes the promoter reactive according to the methylation level. In *ros1* mutants, hypermethylation *of MEMS* is accompanied by increased *ROS1* expression (Lei et al., 2015). Thus, like a thermostat, MEMS is considered to be a “methylstat” that senses and maintains ROS1-dependent methylation in plants (Lei et al., 2015; Williams et al., 2015). Regulation of demethylase gene by sensing methylation level has also been reported in maize (Erhard et al., 2015). Hence, the presence of such “methylstat” is considered to be an essential feature for cytosine methylation dynamics not only in plants but also in animals (Jones et al., 2015; Baylin and Jones, 2016).

### Adenine Methylation

Like cytosine, adenine in DNA can also be methylated by the addition of a CH_3_ group at the N^6^ or N^1^ position (Ratel et al., 2006; Kumar et al., 2018). Methylation of adenine at exocyclic NH_2_ on the sixth position (C^6^) of the purine ring forms N^6^-methyladenine (6-mA). Similarly, methylation of the cyclic N at the first position (N^1^) results in the formation of N^1^-methyladenine (1-mA) due to the presence of endogenous or environmental alkylating agents (Sedgwick et al., 2007). The 6-mA has become a common and well-known player in the regulation of gene expression and defense against phage among the prokaryotes. *AlkB* gene of *E. coli* is considered to be an inducible factor for adaptive response to the environment. An *AlkB* homolog in humans performs a similar function and exhibits significant functional roles (Westbye et al., 2008); therefore, similar factors are expected to be present in plants also. Interestingly, N^7^-methylguanine is also created in the presence of endogenous/environmental alkylating agents. A review by Law and Jacobsen (2010) suggested a certain degree of conservation in the mechanisms for the establishment and maintenance of DNA methylation between animals and plants. While conservation of some of the mechanisms has been confirmed including the role of siRNA in targeted DNA methylation and the role of methylated DNA-binding proteins, several questions regarding adenine methylation/demethylation homeostasis in plants remain to be answered.

Being detected in the lower eukaryotes at the beginning of this century, 6-mA was difficult to be detected in higher eukaryotes probably because of its lesser abundance; hence, earlier considered to be absent in most of the eukaryotes. However, recent advances in high-throughput, highly sensitive techniques, such as deep-sequencing and liquid chromatography coupled with mass spectrometry (LC–MS), have resulted in the detection of 6-mA, its localization in the genome followed by understanding its epigenetic functions in animals and plants (Huang et al., 2015; Liang et al., 2016). Even highly sensitive techniques like mass spectrometry could detect only a few 6-mA per million nucleotides in the genome of animals and plants, which suggests that the turnover (demethylation) rate of 6-mA might be faster. With more intensive studies on modified DNA bases, distribution patterns and possible functions of 6-mA in the animal system are becoming clear day by day; such information is still less known in plants.

The enzymes responsible for conversion of adenine into 6-mA in bacteria (DAMT), *C. elegans* (DAMT-1), *Bombyx mori* and mammals (METTL4), and human (N^6^AMT1) have been well reported (Vanyushin, 2005), but only a little is known about adenine methyltransferase in plants (Li et al., 2019). In green algae *Chlamydomonas*, 6-mA plays an important role in the transcription of genes and nucleosome positioning. In *Chlamydomonas*, adenine-methylome was reported to contain ~85,000 6-mA in AT context mostly in the promoter and linker regions. However, it possesses a low level of 5-mC (Fu et al., 2015). A Mg^2+^/Ca^2+^-dependent N^6^ adenine DNA methyltransferase (wadmtase) purified from wheat coleoptiles showed its potential in generating 6-mA. Wadmtase recognizes TGATCA hexanucleotide, but not GATC tetranucleotide, to methylate adenine (Fedoreyeva and Vanyushin, 2002). This stimulates examination of the presence and the potential role of 6-mA in higher eukaryotes. Analysis of the genomic DNA of *Arabidopsis* revealed the presence of 0.006% (lowest) 6-mA in root and 0.138% (highest) in rosette leaves (Liang et al., 2018). The general distribution of 6-mA in the *Arabidopsis* genome was observed to be near the transcription start site (TSS). The analysis also revealed that 6-mA, particularly those in the TSS region, positively correlates with the expression of the corresponding gene. Furthermore, the changes in 6-mA at different developmental stages of the plant were reported to be associated with gene activation. Although 5-mC and 6-mA both correlate with the transcription of genes in different manners, these epigenetic marks show a certain level of interdependence. Unfortunately, the proteins (readers and erases) that interact with 6-mA in eukaryotes have not yet been characterized.

Analysis of the rice genome revealed about 0.2% of 6-mA, a level similar to that reported in *C. reinhardtii* and *C. elegans* (Fu et al., 2015; Greer et al., 2015). Generally, 6-mA occurs in GAGG context and it was detected in 20% of the genes and 14% of TEs in rice (Zhou et al., 2018). The occurrence of 6-mA was also identified earlier in the GAGG context in *C. elegans*; however, the occurrence of 6-mA in GAGG context in rice is not palindromic, indicating its occurrence only in one strand of DNA (Zhou et al., 2018). While the presence of 6-mA in the promoter causes silencing of the gene, its occurrence in the coding region correlates with activation of the gene. Different possible functions of 6-mA include transcriptional silencing/activation, regulation of transgenerational chromatin functions, and stress response (Liang et al., 2020), as well as in other biological activities like DNA replication and mismatch repair in *E. coli* (Pukkila et al., 1983; Campbell and Kleckner, 1990; Kumar et al., 2018). However, the studies conducted so far report contrasting functions of 6-mA in different eukaryotes.

### Demethylation of Methyladenine

**Figure 3:**
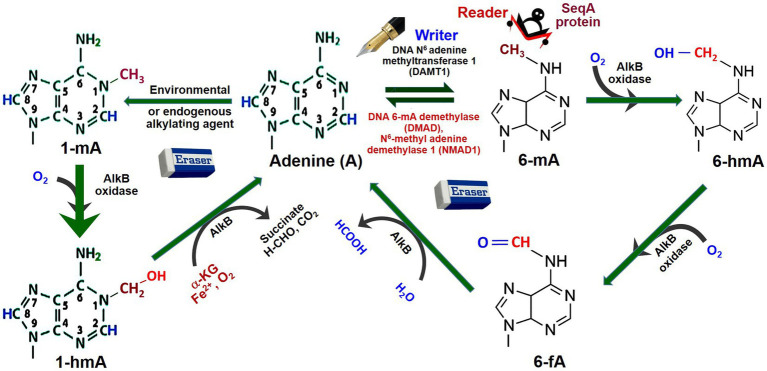
Dynamics of adenine methylation. Adenine (A) gets methylated by the addition of CH_3_ (methyl) group at N^6^ position by DNA adenine methyltransferases 1, the writer, generating N^6^-methyladenine (6-mA). SeqA protein, the reader, specifically binds to hemimethylated 6-mA DNA. The 6-mA gets hydroxylated (–OH) by AlkB oxidase to N^6^-hydroxymethylcytosine (6-hmA). Due to the erasers like DNA 6-mA demethylase (DMAD) or N^6^-methyladenine demethylase-1 (NMAD1), 6-mA gets deaminated back to Adenine. 6-hmA gets demethylated indirectly to Adenine by AlkB oxidase *via* N^6^-hydroxymethyl adenine (6-hmA) and N^6^-formyladenine (6-fA). Adenine may also get methyl adduct to N^1^-methyladenine (1-mA) by environmental/endogenous alkylating agents. Similarly, 1-mA may also get demethylated indirectly by AlkB oxidase to Adenine *via* N^1^-hydroxymethyl adenine (1-hmA). AlkB is one of the prototypic oxidative dealkylation DNA repair enzymes/dioxygenases involved in the removal of alkyl adducts from DNA base by oxidative dealkylation (Redrawn from Kumar et al., 2018).

To some extent, the mechanisms of adenine (de)methylation in animals have been understood. For example, a mutation in DNA methyladenine demethylase (*DMAD*) resulted in the accumulation of 6-mA in *Drosophila*, which revealed its role in adenine methylation/demethylation homeostasis (Zhang et al., 2015). However, an adenine methyltransferase has not been identified in *Drosophila* (Shah et al., 2019). Moreover, oxidation of the attached methyl group at 6-mA by a demethylase (e.g., AlkB dioxygenase) results in its conversion to N^6^-hydroxymethyladenosine (6-hmA) and N^6^-formyladenosine (6-fA), and thus causes demethylation of adenine (Kumar et al., 2018; Figure 3). Studies suggest that AlkB family (Fe^2+^- and α-ketoglutarate-dependent dioxygenases involved in the removal of alkyl adducts from DNA bases by oxidative dealkylation) enzymes are important players in the demethylation of 6-mA (Fu et al., 2015; Iyer et al., 2016). Similarly, 1-mA may also get demethylated by AlkB oxidase and AlkB enzyme *via* N^1^-hydroxymethyladenine (1-hmA).

### Effects of Adenine Methylation

The presence of 6-mA in DNA is recognized by the binding of a specific effector molecule (reader) that may change chromatin conformation and/or transcriptional activity of the gene. Such readers, like SeqA protein, specifically bind to hemimethylated DNA with 6-mA. For example, polycomb proteins were reported as the coordinator between the accumulation of 6-mA and deactivated DMAD for transcriptionally repressing the gene (Yao et al., 2018). When present in the promoter, 6-mA generally represses the expression of the gene, but it may also function as an activator of the transcription process. These suggest that (de)methylation of adenine and cytosine takes place in a dynamic, coordinated, and context-specific manner. Therefore, it would be interesting to understand the interaction among the epimarks to investigate the complexity of epigenetic codons, which might help to answer several biological enigmas (Kumar, 2017; Kumar et al., 2018). A comprehensive understanding of such modifications and their functions in epigenetic regulation of gene expression would be essential for epigenetic manipulation of desirable traits in plants and animals (Kumar, 2019b).

## Functions of Methylated DNA Bases

DNA base modifications, particularly cytosine methylation, were initially considered as a host defense mechanism in prokaryotes. Later on, it was found to play several vital functions in eukaryotes, mostly as a defense mechanism against jumping TEs to maintain genome integrity over the generations (Zhang et al., 2011). Over the last two decades, epigenetic changes in the plant genome have been reported during various developmental processes and environmental stresses (Bartels et al., 2018). Methylcytosine in the promoter region was reported to repress transcription of the gene by affecting the binding of TFs and by forming repressive-chromatin structures due to the interaction between methylated DNA-binding proteins (Bird, 2002). Regulatory flexibility is a characteristic feature of epigenetic mechanisms, particularly in response to environmental factors. Similarly, gene/genome imprinting (preferential expression of the gene/genome coming either from male or female parents) is also regulated through epigenetic mechanisms. Imprinted genes are silenced by DNA and/or histone modifications. Demethylation of the maternal genome and activation of the genes in endosperm have been reported in *Arabidopsis*, rice (Hsieh et al., 2009; Luo et al., 2011; Rodrigues et al., 2013), and maize (Waters et al., 2011). Knockout of *ALKBH1* (a 6-mA demethylase) resulted in a higher 6-mA level and early flowering in rice (Zhou et al., 2018), suggesting that 6-mA plays an important role in reproductive development in rice. Nucleosome remodeler (e.g., DDM1) affects 5-mC content in plants (Tan et al., 2016). Decreased 6-mA content and no change in 5-mC level were reported in CRISPR/Cas9 *ddm1a*/*ddm1b* double mutants showing dwarfing and decreased seed-setting (Zhang et al., 2018b) suggesting that 6-mA is involved in vegetative and reproductive developments in rice. Thus, DNA base and histone protein modifications in combination with the nonhistone proteins define accessibility of the genes to help regulate their expression.

### Source of Diversity and Heritable Variations

Some of the epigenetic changes may persist even after the reversal of the conditions that caused such changes, and some of them may inherit to the next generation as epigenetic alleles (epialleles). Such heritable epialleles are being considered as an additional source of diversity, which may be utilized in breeding programs, particularly in those crops where genetic diversity is reported to be scarce. The creation of natural epialleles is much faster than that of alleles due to natural genetic mutation; however, the reversal rate of epialleles is also higher. Even then, epigenetics is considered to create more heritable epialleles and helps in the evolution process. Reports suggest that environmentally induced epigenetic changes in plants may be mitotically stable and meiotically inherited. Therefore, the emphasis is now given to such epigenetic changes as a source of variation. Transcriptional activation of Tos17 retrotransposon (RT) during tissue culture in rice was reported earlier, which gets repressed on plant regeneration (Liu et al., 2004). Studies demonstrate that activation of the transcription process and transposition of the RT in tissue-cultured calli are controlled through DNA hypomethylation (Cheng et al., 2006; Ding et al., 2007). Later on, it was reported that RT is demethylated by DNA glycosylase/lyase which promotes its movement during tissue culture in rice (La et al., 2011).

RdDM pathway was reported to respond to the environmental stimuli, which triggers epigenetic changes at particular loci toward the generation of heritable epialleles (Manning et al., 2006; Verhoeven et al., 2010). However, the importance of epialleles in crop breeding would require determining the extent of variation in epimarks among the individuals, the extent to which the epimarks affect the phenotype, and the heritability of the epimark-linked superior phenotypes. Moreover, several technical challenges in estimating the epigenetic variations and the level of epimark-associated phenotypic diversity do exist. With the continuously increasing understating of epigenomics, it is expected that our efficiency of exploiting epigenomic variability and deploying epigenome editing in crop improvement would become better and will have a significant impact on food security.

### Regulation of Gene Expression

DNA modification in different cells/tissues is dynamically regulated during plant growth, development, and under varying environmental conditions. This indicates the important roles of DNA modifications in the regulation of gene expression and physiology. Base modifications occurring in a promoter, in the nearby regions, and/or within the gene-body, might affect the gene expression. Generally, DNA base modifications repress transcription of the gene; however, in certain cases, this may also promote transcription of the gene. Such an example is cytosine methylation in the promoter of ROS1 which enhances its transcription in *Arabidopsis* (Williams et al., 2015). Base modification may strengthen the binding of certain transcription activators, or it may inhibit the binding of transcription repressor. However, the exact mechanisms of regulating gene expression by DNA methylation in the promoter region and the gene-body are not yet clear. Since, only 5% of the genes in *Arabidopsis* are methylated in the promoter region, which indicates that DNA methylation is not the sole epigenetic regulatory mechanism for controlling the expression of genes (Zhang et al., 2018a; Kumar and Mohapatra, 2021). Crop plants with a large genome size possess a higher number of TEs, and many of them are closer to genes affecting their expression. Thus, DNA modification plays a significant role in controlling the expression of the gene in crop plants compared to that in *Arabidopsis* which contains a limited number of TEs. However, DNA demethylase targets TEs present in the promoter to regulate stress-responsive genes (Le et al., 2014). That is why DNA methylation mutants in crop plants have been reported to have severe growth/developmental defects or lethal effects (Wei et al., 2014; Liu et al., 2015; Lang et al., 2017).

In *Arabidopsis*, about one-third of the genes are methylated in the gene body (Zhang et al., 2006). In general, TEs and repeat regions are heavily methylated in all cytosine contexts, but gene body methylation sparsely occurs in non-CG context (Zhang et al., 2006; Cokus et al., 2008; Takuno and Gaut, 2013). Gene body methylation occurs in the transcribed/coding region between the transcription start and termination sites (Bewick and Schmitz, 2017). Some of the introns in a gene may harbor TE or repeat elements, which are hypermethylated in all cytosine contexts and regulate mRNA processing. Loss of DNA methylation from a retrotransposon present in an intron of the homeotic gene was reported to cause alternate splicing and premature termination of the transcript. The role of 6-mA in the regulation of gene expression appears to be conserved among the plants. 6-mA occurs in the gene-body of a transcriptionally active gene (Liang et al., 2018; Zhang et al., 2018b; Zhou et al., 2018). 6-mA and 5-mC sites might show overlap, and 6-mA-containing genes may show a high degree of nucleosome arrays (Zhou et al., 2018). This indicates that 6-mA provides an additional layer of the epigenetic regulatory mechanism of gene expression or it works along with the other epigenetic markers.

### Transposon Silencing and Genome Stability

Active TEs threaten genome stability/integrity due to the jumping of transposons or repeated insertion of retrotransposons. Heterochromatins as well as transposon-or repeat-containing euchromatic regions in *Arabidopsis* are hypermethylated in all cytosine contexts (Cokus et al., 2008). CHH methylation in smaller transposons and at the ends of long transposons is established by the RdDM pathway, while it is taken care of by DDM1 and catalyzed by CMT2 at the internal positions of heterochromatin and long transposons (Zemach et al., 2013; Stroud et al., 2014). The active genes and inactive transposons in the maize genome are separated by RdDM-dependent hypermethylated CHH islands. Any loss of methylated CHH island leads to transcriptional activation of the transposon, suggesting that RdDM is needed to keep the transposons silenced (Li et al., 2015). Transposon reactivation due to DNA demethylation was observed in only a few *Arabidopsis* mutants, whereas *met1*-*cmt3* double mutants or *ddm1* mutants showed hypomethylation in CG and CHG contexts, and increased transposition of TEs (Mirouze et al., 2009; Tsukahara et al., 2009). DNA glycosylase/lyase 701 (a ROS1 homolog) controls the movement of retrotransposon Tos17 in rice, which indicates that DNA modification regulates transposon activity (La et al., 2011). DNA methylation also influences chromosomal interactions. In *Arabidopsis*, all the five chromosomes were reported to interact with each other forming a structure known as KNOT (Grob et al., 2014). Moreover, the chromosomal regions involved in the formation of KNOT are comprised of interactive heterochromatin islands (IHIs) containing several transposons (Feng et al., 2014; Grob et al., 2014). The KNOT engaged element represents a preferred landing site for TEs, which may be a part of the defense mechanisms for genome stability. Increased chromosome interaction between the Pol V-dependent methylation and the genes repressed by RdDM was recently reported (Rowley et al., 2017). This indicates that even chromosomal interactions might have regulatory functions in gene expression. Several studies suggest a wide variation in DNA methylation among different cells, tissues, organs, and species. The variation in DNA methylation level, GC content, and chromatin architecture among different species do not correlate with the genome size and thus serve as the source of diversity.

### Genome Imprinting and Heterosis

FIS2, FWA, and MEA are some of the well-characterized genes responsible for genome imprinting in plants. While the allele from one parent is expressed, the allele from the other parent is silenced. This is known as genome imprinting (Gehring et al., 2006; Jullien et al., 2006). In flowering plants, megaspore mother cell (MMC) undergoes meiosis to form female reproductive organs. Similarly, the microspore mother cell (MiMC) undergoes meiosis to form male reproductive organs. Both MMC and MiMC undergo large-scale chromatin changes, including heterochromatin decondensation, during cell specification indicating a highly active transcriptional activity (She and Baroux, 2015). During MMC specification, CHH methylation transiently decreases and then gets restored but CG methylation remains stable. While methylation at CG and CHG contexts is maintained by MET1 and CMT3, respectively, CHH methylation is maintained by CMT2 or by the RdDM pathway (Gehring, 2019). Recently, DNA methylation was profiled in the MiMC of *Arabidopsis*, wherein high levels of CG and CHG methylation but low level of CHH methylation were reported (Walker et al., 2018).

**Figure 4:**
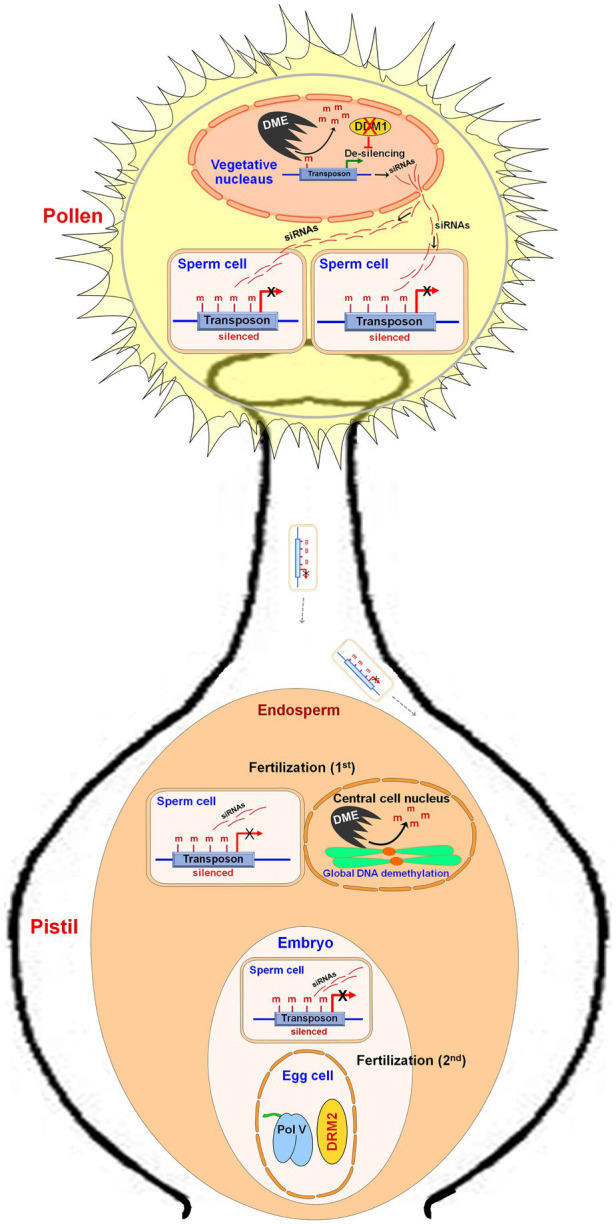
Role of DNA methylation during reproductive development in plants. During male gametogenesis in pollen, the transposons in the vegetative cell are de-silenced due to DNA demethylation by Demeter (DME) and by downregulated expression of Decreased DNA Methylation 1 (DDM1, a chromatin remodeler). Transposon-generated transcripts are converted into siRNAs, which enter into the sperm cells and cause silencing of transposon through DNA methylation. On pollination, one of the sperm cells fertilizes a female gamete (central cell) and forms the endosperm, wherein reinforced CHH methylation at transposons (in the male genome) and DME-mediated global DNA demethylation (in the female genomes) are observed. Another sperm cell fertilizes the egg cell to form an embryo, where reinforced CHH methylation in the male genome but domain rearranged methylase 2 (DRM2) and RNA polymerase V (Pol V) derived RdDM pathway in the female genome are observed.

DME expression in the central cell before fertilization was reported to cause extensive demethylation of the maternal genome (Gehring et al., 2009; Hsieh et al., 2011) which causes the expression of the genes from the maternal genome. Zhang et al. (2011) carried out MSAP analysis and reported hypomethylation in the endosperm of *Sorghum bicolor* because of demethylation in the CG context. Genome-wide demethylation of TEs was also reported in the endosperm (Gehring et al., 2009; Hsieh et al., 2009). During male gametogenesis in plants, the transposons present in vegetative/companion cells are derepressed by transcription activator DME-mediated DNA demethylation and downregulated expression of a chromatin remodeler DDM1 (Lippman et al., 2004; Zhang et al., 2016). In the vegetative cell, the transcripts generated from TEs are processed into siRNAs and enter sperm cells to reinforce transposon silencing *via* DNA methylation (Martínez et al., 2016). In a double-fertilization system, one of the two sperm cells of pollen fertilizes the central cell of the female gametophyte (first fertilization) while the other fertilizes the egg cell (second fertilization), which produce endosperm and embryo, respectively. A sperm cell fertilizes with a central cell of the female gamete to form the endosperm wherein global demethylation, but reinforced CHH methylation at TEs, is observed (Ibarra et al., 2012). Another sperm cell fertilizes the egg cell to produce the embryo where the RdDM pathway maintains methylation (Figure 4). In *Arabidopsis* as well as in rice, the endosperm displays global DNA hypomethylation compared to that in the embryos (Gehring et al., 2009; Hsieh et al., 2009; Ibarra et al., 2012). Imprinting of endosperm is schemed by differential DNA methylation of the maternal and paternal genomes together with the polycomb group of genes (Hsieh et al., 2011). The maternal genomes of endosperm are less methylated (particularly in CG context) compared to that of the paternal genome (Gehring et al., 2009; Hsieh et al., 2009; Zhang et al., 2014; Klosinska et al., 2016). Certain maternally expressed genes (MEGs), for example, MEDEA in *Arabidopsis*, from the paternal genome are silenced due to repressive histone (H3K27me3) modification rather than by DNA methylation (Gehring et al., 2006; Jullien et al., 2006). In maize, the endosperm-specific MEGs are associated with H3K4me3 modification, while paternal alleles are suppressed by hypermethylation near the TSSs (Dong et al., 2017). Thus, the set of imprinted genes show that imprinting is a major epigenetic phenomenon affecting endosperm development in plants. RdDM pathway was reported to regulate parental gene imprinting at several loci in *Arabidopsis* (Vu et al., 2013). Thus, manipulation in genome imprinting through epigenome editing might help to develop a superior endosperm for improvements in seed crops (Berger, 2003; Kumar, 2019a).

Evidence suggests that F_1_ hybrids are hypomethylated compared to their parental inbreds (Kovacevic et al., 2005). RNA amount polymorphism and protein amount polymorphism data in maize indicate that quantitative variations in the expression of certain loci might be responsible for the heterosis observed in the F_1_ hybrid. Repeated selfing during the development of inbreds, giving more emphasis on combining ability, might result in a gradual accumulation of methylated loci (Kumar et al., 2017b). This gets repatterned and/or released when the inbreds are crossed to develop a hybrid. Further, understanding the epigenetic regulation of embryo development might uncover the mysteries of apomixis (asexual reproduction through seeds) in a plant (Koltunow and Grossniklaus, 2003; Kumar, 2017; Rathore et al., 2020). If apomixis is successfully incorporated in commercial seed crops, heterosis can be preserved over the generations to overcome the current limitations of hybrid seed production.

### Fruit Ripening, Seed Development, and Germination

DNA hypomethylation is reported to be a general feature at the promoter of many fruit ripening-associated genes as they contain binding sites for the ripening-associated transcription factors (Zhong et al., 2013; Lang et al., 2017). Ripening inhibitor binds to the methylated promoter of ripening genes to suppress the expression of the genes. The expression of DML2 (DNA demethylase) increases dramatically during the ripening of tomatoes. Active demethylation is required not only to activate ripening genes but also to suppress the ripening-inhibitor genes (Liu et al., 2015; Lang et al., 2017). Hypermethylation at CHH context in developing apple fruit, compared to that in the leaf, has been reported. A correlation between DNA hypomethylation and the smaller size of the fruit has also been reported (Daccord et al., 2017). Anthocyanin content in apple fruits has been reported to be negatively correlated with DNA methylation level at the promoter of MYB10 gene (Telias et al., 2011; El-Sharkawy et al., 2015).

Seed development is an essential process for food quality and productivity. During seed development in soybean, CHH methylation was reported to increase from 6% at the early stage to 11% in the late stage (An et al., 2017; Lin et al., 2017). Methylome analysis at the globular stage and seed germination in *Arabidopsis* and soybean showed a significant increase in methylation at CHH context (Lin et al., 2017). Twenty-five genes were observed to be differentially methylated during rice seed development, and endosperm cellularization was reported to be regulated by methylation dynamics (Xing et al., 2015). In *Brassica rapa*, a mutation in the RdDM pathway resulted in reproductive defects, which suggests that DNA modification is necessary for seed development (Grover et al., 2018). The maternal allele of components in the RdDM pathway was reported to be required for seed development in *Brassica rapa* (Grover et al., 2018). Demethylation of a retroelement (RE) Gy163 was observed to be associated with apomictic seed development in *Cenchrus ciliaris*. The RE Ty3-gypsy was found to be differentially methylated/expressed in the reproductive tissues of apomictic and sexual plants. Hypomethylation was observed in CHH context in reproductive (aposporous initial and mature embryo sac) tissues of apomictic plants, which was directly correlated with the activity of the RE (Rathore et al., 2020). Thus, epigenetic regulation of seed development appears to be a common process in different plant species.

### Tolerance to Abiotic Stress

**Figure 5:**
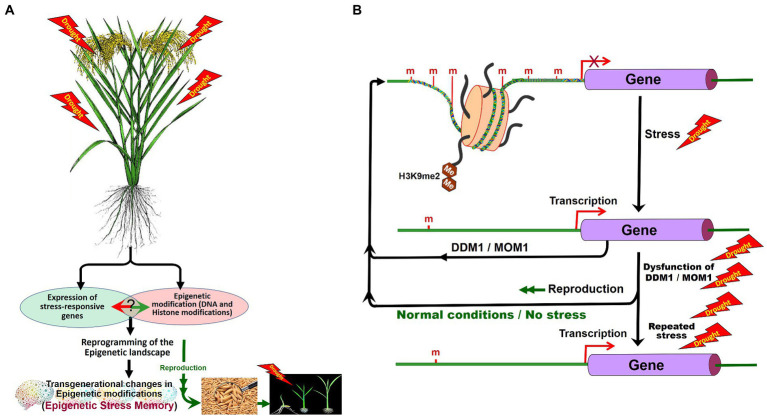
Role of DNA methylation in stress tolerance and epigenetic stress memory in plants. Abiotic stresses may alter the expression of the stress-responsive genes and cause DNA base modifications. In stressed plants, epigenetic modifications reprogram the epigenetic landscape and some of the epigenetic changes may be inherited **(A)**. Expression of stress-responsive genes may also cause changes in epigenetic modifications. After the stress, during the recovery period, Morpheus Molecule 1 (MOM1) and DDM1 erase the stress-induced epigenetic marks. Inheritance of stress-induced epigenetic marks/de-silencing of genes can be seen in plants repeatedly exposed to the tress due to the dysfunction of DDM1 and MOM1 **(B)**. H3K9me2, dimethylated histone H3 lysine 9 (Modified from Zhang et al., 2018a).

Abiotic stresses have been reported to cause alterations in DNA methylation in plants. Heat stress-induced accumulation of *ONSEN* retrotransposon was observed in *Arabidopsis* mutants impaired in the biogenesis of siRNAs (Ito et al., 2011). P5CS and *δ-OAT* genes were reported to be demethylated under osmotic stress in mother plants, but they restore methylation in the next generation under normal growth conditions (Zhang et al., 2013b), suggesting that epigenetic changes regulate the expression of the genes (Figure 5A). TEs have been reported to affect the expression of genes through their *cis-or trans*-regulatory elements, or even through serving as targets of epigenetic modifications (Seymour et al., 2014; Stuart et al., 2016; Wei and Cao, 2016). TEs being one of the targets of epigenetic machinery for controlling their activity as well as that of the nearby genes, they play important roles in the adaptation of plants to the changing global climate (Li et al., 2018b). Masuta et al., (2017) analyzed transcriptional and transpositional activation of *ONSEN* and observed heat stress-induced transposition of *ONSEN* during tissue culture. Heat stress response of soybean root showed a marginal (<10%) decrease in methylation; however, a significant change in the CHH context and TEs was observed (Hossain et al., 2017).

Several early studies on abiotic stresses indicate stress-induced DNA (de)methylation of stress-associated genes. Phosphate (Pi) starvation in rice was reported to cause more than 100 differentially methylated regions (DMRs) due to hypermethylation in CHH context mainly in the transposons near Pi-starvation-induced genes (Secco et al., 2015). Salt stress-induced changes in DNA methylation were reported to be partly inherited over the generations in *Arabidopsis*, especially through female gametes (Wibowo et al., 2016). Suppressor of DRM1, DRM2, and CMT3 (*SDC*) gene was reported to be silenced through DNA methylation of the promoter in vegetative tissues. Stress-induced activation of *SDC* due to repeated stress was also reported (Sanchez and Paszkowski, 2014). The RdDM pathway has been reported to alter TEs activity and gene expression involved in abiotic and biotic stress responses, plant development, and intercellular communication.

Recent studies suggest that 6-mA plays an important role in regulating gene expression in plants under environmental stress. 6-mA level showed dynamic changes in rice under heat, cold, and salt stress (Zhang et al., 2018b). Under heat stress, 6-mA level in the heat-tolerant rice genotype was 2.6-fold greater than that in the heat-sensitive rice genotype. 6-mA content in a heat stress master transcriptional regulator (heat shock transcription factor A1) exhibited a significant increase in heat-tolerant genotype than in heat-sensitive rice genotype. Decreased 6-mA level in HsfA1 repressor HSP70 in heat-tolerant genotype improves tolerance to heat stress (Zhang et al., 2018b). DDM1 and Morpheus molecule 1 (MOM1) in *Arabidopsis* were reported to be responsible for erasing stress-induced epigenetic marks after the stress. In double mutants for *ddm1–mom1*, stress-induced epigenetic marks are transmitted to the progeny, whereas in a single mutant either for *ddm1* or for *mom1*, stress-induced epigenetic marks are not inherited. This indicates that MOM1 and DDM1 function in checking the inheritance of stress-induced epimarks (epigenetic stress memory; Iwasaki and Paszkowski, 2014). The stress-induced epigenetic memory may also be lost due to the passive demethylation process, if the progenies are grown under normal (without stress) conditions (Zhang et al., 2018a; Figure 5B).

### Tolerance to Biotic Stress

In addition to the abiotic stresses, plants are also challenged by various biotic stresses like insect pests and diseases. Several studies have established the role of epigenetic variations in plant-microbe interaction mainly through gene regulation (Boyko and Kovalchuk, 2011; Diez et al., 2014; Zhu et al., 2015; Espinas et al., 2016). Another mechanism affecting the susceptibility of plants to pathogens has been reported which includes methylation of plant and viral genome (Wang et al., 2012; Sharma et al., 2013; Baulcombe and Dean, 2014). The importance of DNA methylation is emerging in transcriptional regulation of the virus-induced genes (Sahu et al., 2013; Ding and Wang, 2015; Wang et al., 2018a). Moreover, plants have evolved several defense mechanisms to cope up with viral infection mainly *via* siRNA-mediated antiviral silencing (Pumplin and Voinnet, 2013; Sharma et al., 2013). The siRNA cleaves/represses translation of the target mRNA and thus causes posttranscriptional gene silencing. Methylation of DNA base and/or histone protein causes gene silencing at the transcriptional level, which is known as transcriptional gene silencing (TGS). While DNA viruses are targeted through TGS to restrict their proliferation (Rodríguez-Negrete et al., 2009), RNA viruses are not influenced by DNA methylation. However, methylation of RNA bases, e.g., N^6^-methyladenosine (m^6^A; Gokhale et al., 2016; Kumar and Mohapatra, 2021), controls viral replication as well as the interaction between virus and host (Brocard et al., 2017; Dang et al., 2019). Hundreds of DMRs were identified to be influenced by a viral infection in tobacco, several of which were reported to be associated with gene expression to regulate host antiviral defense (Wang et al., 2018b). Plants use methylation of viral genomic DNA to restrict its replication, while virus encodes viral suppressor proteins to protect it from getting methylated (Raja et al., 2010). The viral suppressors might interfere with the host methylation pathways to benefit the virus. A significant variation in methylation at CHH context was observed in *Arabidopsis* in response to different biotic stresses (Slaughter et al., 2012); however, the role of such alteration in DNA methylation in priming the plants against pests/diseases is not known (Wang et al., 2019). Interestingly, evidence shows that stress priming changes the epigenetic profile of plants which may improve the stress tolerance ability of the plant (Lopez Sanchez et al., 2016; Lämke and Bäeurle, 2017; Varotto et al., 2020).

### Stress Memory and Adaptation

It has been shown that plants can remember past environmental stress and use the memories to respond rapidly to the stress when it recurs (Hilker and Schmülling, 2019). Plants have evolved various sensing and signaling mechanisms to respond appropriately to stress. Evidence gathered through various studies indicates that epigenetic variations are necessary as a part of the stress memories and adaptation in plants (Thiebaut et al., 2019). Moreover, plants have also evolved certain mechanisms by which they can memorize the events of past stress and trigger the responses to respond quickly/strongly to recurrent stress. Memorizing the events requires the storage of information, which might occur in plants (without a nervous system) in the form of chromatin architecture, transcription factor, posttranslational modifications, phytohormones, metabolites, etc., involved in the stress management (Xing et al., 2018; Hilker and Schmülling, 2019). The role of epigenetic variations in stress priming/memory is being studied (Crisp et al., 2016; Lämke and Baeurle, 2017; He and Li, 2018). Alterations to H3K4me3, which is a transcription activation mark, suggest that this epigenetic mark plays a role in transcription memory as it is enriched by drought stress and maintained at a higher level during the rehydration process (Kim et al., 2012). The small-RNA may also play a role in stress memory, as reported in the case of repeated drought stress to *Arabidopsis* (Ding et al., 2012). *Arabidopsis* plants with a mutation in *met1* were observed to be resistant to low humidity stress (Tricker et al., 2012). Moreover, a higher stomatal index was detected in *Arabidopsis* mutants for *dcl3* or *rdr6* under low humidity, indicating that RdDM pathway plays a role in remembering the stress. Transgenerational inheritance of epigenetic marks involves passing of the epigenetic changes through the germline without getting erased by the surveillance mechanisms (Lange and Schneider, 2010). Accumulating evidence indicates that short-term memory and transgenerational memories rely on epigenetic mechanisms; hence, they can be utilized in developing climate-smart crop varieties. However, many questions regarding the role of epigenetic marks in keeping the stress memory, their persistence, and stability during mitosis are still unanswered.

Many (~70%) stress-induced epigenetic alterations revert to the original state once the stress disappears, but a part of the epigenetic modifications might be carried forward as epigenetic stress memory (Crisp et al., 2016; Kumar, 2018). Therefore, utilizing such epialleles in breeding programs is still a challenging task because of the transgenerational stability of the environment-induced epigenetic alterations. Zheng et al. (2017) reported a high proportion of drought-induced DNA methylation in rice and maintenance of their pattern in successive generations under drought stress. Such findings suggested that epigenetic modifications can be utilized in improving stress responses of crop plants. Thus, epigenetic manipulation may become an efficient tool for crop improvement, as appropriate strategies are becoming available for the modulation of DNA methylation using chemicals or by genetic means, followed by the forward or reverse epigenetic approach. However, appropriate strategies would be required to ensure retention of the transferred/introduced epialleles in the new genetic environment. Moreover, epigenome editing may help achieve the desired changes and adaptive advantages without entering into the controversy of genetic engineering (Gallego-Bartolomé et al., 2018; Kumar, 2019a).

## Conclusion and Future Perspectives

Much progress has been made in our understanding of epigenetic regulation of gene expression, particularly in model plants like *Arabidopsis* and rice. Proteins and enzymes involved in DNA and histone modifications in plants are being characterized continuously. However, we still know a little about the components controlling targeted DNA (de)methylation during the developmental process and environmental stress. Does DNA modification interplay with other epigenetic marks and affect chromatin conformation are some of the enigmatic questions that need to be answered for a better understanding of epigenomics. Increasing focus on 6-mA as an additional epigenetic mark raises several questions like (1) which AlkB family protein acts as an eraser of 6-mA? (2) Whether/how does adenine methylation-and demethylation machinery interact with histone modification and transcription machinery? (3) Moreover, the readers of the 6-mA mark are yet to be discovered. DNA methylation pattern of different plant species varies because every species possesses a different set of DNA methyltransferases (MET1, CMT2, CMT3, DRM1, and DRM2), demethylases (DME, ROS1, DML2, and DML3), and RdDM pathway to (de)methylate the TEs, repeats, and genes to switch them on/off (Bartels et al., 2018).

Toward epigenetic manipulation, a catalytically inactive SpdCas9 fused with (de)methylase (SpdCas9-Tet1 and SpdCas9-Dnmt3a) was reported to be useful in epigenome editing in a site-specific manner in mammalian cells (Liu et al., 2016). In a recent study, on the development of the tool for targeted DNA demethylation in plants, Gallego-Bartolomé et al. (2018) reported efficient and targeted demethylation with minimal off-target effects in plant. This can also be used to answer some of the basic questions in epigenomics, to develop new strategies for modulating gene expression, and to create a new epiallele for the desired trait in plants. Gallego-Bartolomé et al. (2018) utilized fusion of the catalytic domain of human demethylase (TET1cd) with an artificial zinc finger targeting promoter of the *Flowering Wageningen* (*FWA*) resulting in efficient and targeted demethylation, *FWA* upregulation, and heritable late-flowering phenotype in *Arabidopsis*. Recently (Taghbalout et al., 2016) used Casilio-ME for RNA-guided editing of 5-mC by targeting TET1 to specific genomic sites, and co-delivery of TET1 and other protein factors, to activate methylation-silenced genes. Similarly, Devesa-Guerra et al. (2020) fused the catalytic domain of ROS1 5-mC DNA glycosylase with dCas9 and reported that dCas9-ROS1 (but not the dCas9-TET1) can reactivate methylation-silenced genes by active demethylation. With the advances in epigenomic tools and techniques, it can be expected that very soon we might be able to use epigenome editing to modulate phenotypic plasticity of plants (Kumar, 2019a) toward developing climate-smart crops for sustainable agriculture.

## Author Contributions

SK and TM conceived the review. SK prepared the preliminary draft. SK and TM revised the manuscript and approved the final draft. All authors contributed to the article and approved the submitted version.

### Conflict of Interest

The authors declare that the research was conducted in the absence of any commercial or financial relationships that could be construed as a potential conflict of interest.

## References

[ref1] Almeida K. H. Sobol R. W. (2007). A unified view of base excision repair: lesion-dependent protein complexes regulated by post-translational modification. DNA Repair 6, 695–711. 10.1016/j.dnarep.2007.01.009, PMID: 17337257 PMC1995033

[ref2] An Y. C. Goettel W. Han Q. Bartels A. Liu Z. Xiao W. (2017). Dynamic changes of genome-wide DNA methylation during soybean seed development. Sci. Rep. 7, 1–14. 10.1038/s41598-017-12510-4, PMID: 28947812 PMC5613027

[ref3] Bartels A. Han Q. Nair P. Stacey L. Gaynier H. Mosley M.. (2018). Dynamic DNA methylation in plant growth and development. Int. J. Mol. Sci. 19:2144. 10.3390/ijms19072144, PMID: 30041459 PMC6073778

[ref4] Baulcombe D. C. Dean C. (2014). Epigenetic regulation in plant responses to the environment. Cold Spring Harbor Perspect. Biol 6:a019471. 10.1101/cshperspect.a019471, PMID: 25183832 PMC4142964

[ref5] Baylin S. B. Jones P. A. (2016). Epigenetic determinants of cancer. Cold Spring Harb. Perspect. Biol. 8:a019505. 10.1101/cshperspect.a019505, PMID: 27194046 PMC5008069

[ref6] Bell A. C. Felsenfeld G. (2000). Methylation of a CTCF-dependent boundary controls imprinted expression of the *Igf2* gene. Nature 405, 482–485. 10.1038/35013100, PMID: 10839546

[ref7] Berdis A. J. Lee I. Coward J. K. Stephens C. Wright R. Shapiro L.. (1998). A cell cycle-regulated adenine DNA methyltransferase from *Caulobacter crescentus* processively methylates GANTC sites on hemimethylated DNA. Proc. Natl. Acad. Sci. U. S. A. 95, 2874–2879. 10.1073/pnas.95.6.2874, PMID: 9501183 PMC19662

[ref8] Berger F. (2003). Endosperm: the crossroad of seed development. Curr. Opin. Plant Biol. 6, 42–50. 10.1016/S1369526602000043, PMID: 12495750

[ref9] Bewick A. J. Schmitz R. J. (2017). Gene body DNA methylation in plants. Curr. Opin. Plant Biol. 36, 103–110. 10.1016/j.pbi.2016.12.007, PMID: 28258985 PMC5413422

[ref10] Bies-Etheve N. Pontier D. Lahmy S. Picart C. Vega D. Cooke R.. (2009). RNA-directed DNA methylation requires an AGO4-interacting member of the SPT5 elongation factor family. EMBO Rep. 10, 649–654. 10.1038/embor.2009.31, PMID: 19343051 PMC2711833

[ref11] Bird A. P. (2002). DNA methylation patterns and epigenetic memory. Genes Dev. 16, 6–21. 10.1101/gad.947102, PMID: 11782440

[ref12] Boyko A. Kovalchuk I. (2011). Genetic and epigenetic effects of plant–pathogen interactions: an evolutionary perspective. Mol. Plant 4, 1014–1023. 10.1093/mp/ssr022, PMID: 21459830

[ref13] Brocard M. Ruggieri A. Locker N. (2017). m^6^A RNA methylation, a new hallmark in virus–host interactions. J. Gen. Virol. 98, 2207–2214. 10.1099/jgv.0.000910, PMID: 28869001

[ref002] Calarco J. P. Borges F. Donoghue M. T. Van Ex F. Jullien P. E. Lopes T. D.. (2012). Reprogramming of DNA methylation in pollen guides epigenetic inheritance via small RNA. Cell 151, 194–205. 10.1016/j.cell.2012.09.001, PMID: 23000270 PMC3697483

[ref14] Campbell J. L. Kleckner N. (1990). *E. coli* oriC and the *dnaA* gene promoter are sequestered from dam methyltransferase following the passage of the chromosomal replication fork. Cell 62, 967–979. 10.1016/0092-8674(90)90271-F, PMID: 1697508

[ref15] Cheng C. Diagen M. Hirochika H. (2006). Epigenetic regulation of the rice retrotransposon Tos17. Mol. Gen. Genomics. 276, 378–390. 10.1007/s00438-006-0141-9, PMID: 16821043

[ref16] Cokus S. J. Feng S. Zhang X. Chen Z. Merriman B. Haudenschild C. D.. (2008). Shotgun bisulphite sequencing of the *Arabidopsis* genome reveals DNA methylation patterning. Nature 452, 215–219. 10.1038/nature06745, PMID: 18278030 PMC2377394

[ref17] Crisp P. A. Ganguly D. Eichten S. R. Borevitz J. O. Pogson B. J. (2016). Reconsidering plant memory: intersections between stress recovery, RNA turnover, and epigenetics. Sci. Adv. 2:e1501340. 10.1126/sciadv.1501340, PMID: 26989783 PMC4788475

[ref18] Daccord N. Celton J.-M. Linsmith G. Becker B. Choisne N. Schijlen E.. (2017). High-quality de novo assembly of the apple genome and methylome dynamics of early fruit development. Nat. Genet. 49, 1099–1106. 10.1038/ng.3886, PMID: 28581499

[ref19] Dang W. Xie Y. Cao P. Xin S. Wang J. Li S.. (2019). N^6^-methyladenosine and viral infection. Front. Microbiol. 10:417. 10.3389/fmicb.2019.00417, PMID: 30891023 PMC6413633

[ref20] Devesa-Guerra I. Morales-Ruiz T. Pérez-Roldán J. Parrilla-Doblas J. T. Dorado-León M. García-Ortiz M. V.. (2020). DNA methylation editing by CRISPR-guided excision of 5-methylcytosine. J. Mol. Biol. 432, 2204–2216. 10.1016/j.jmb.2020.02.007, PMID: 32087201

[ref21] Diez C. M. Roessler K. Gaut B. S. (2014). Epigenetics and plant genome evolution. Curr. Opin. Plant Biol. 18, 1–8. 10.1016/j.pbi.2013.11.017, PMID: 24424204

[ref22] Ding B. Wang G. L. (2015). Chromatin versus pathogens: the function of epigenetics in plant immunity. Front. Plant Sci. 6:675. 10.3389/fpls.2015.00675, PMID: 26388882 PMC4557108

[ref23] Ding Y. Fromm M. Avramova Z. (2012). Multiple exposures to drought ‘train’ transcriptional responses in *Arabidopsis*. Nat. Commun. 3, 1–9. 10.1038/ncomms1732, PMID: 22415831

[ref24] Ding Y. Wang X. Su L. Zhai J. X. Cao S. Y. Zhang D. F.. (2007). SDG714, a histone H3K9 methyltransferase, is involved in Tos17 DNA methylation and transposition in rice. Plant Cell 19, 9–22. 10.1105/tpc.106.048124, PMID: 17259261 PMC1820975

[ref25] Dong X. M. Zhang M. Chen J. Peng L. Zhang N. Wang X.. (2017). Dynamic and antagonistic allele-specific epigenetic modifications controlling the expression of imprinted genes in maize endosperm. Mol. Plant 10, 442–455. 10.1016/j.molp.2016.10.007, PMID: 27793787

[ref26] Du J. M. Johnson L. M. Groth M. Feng S. Hale C. J. Li S.. (2014). Mechanism of DNA methylation-directed histone methylation by KRYPTONITE. Mol. Cell 55, 495–504. 10.1016/j.molcel.2014.06.009, PMID: 25018018 PMC4127122

[ref27] Du J. M. Zhong X. Bernatavichute Y. V. Stroud H. Feng S. Caro E.. (2012). Dual binding of chromomethylase domains to H3K9me2-containing nucleosomes directs DNA methylation in plants. Cell 151, 167–180. 10.1016/j.cell.2012.07.034, PMID: 23021223 PMC3471781

[ref28] Duan C. G. Wang X. Xie S. Pan L. Miki D. Tang K.. (2017). A pair of transposon-derived proteins function in a histone acetyltransferase complex for active DNA demethylation. Cell Res. 27, 226–240. 10.1038/cr.2016.147, PMID: 27934869 PMC5339849

[ref29] Ebbs M. L. Bender J. (2006). Locus-specific control of DNA methylation by the *Arabidopsis* SUVH5 histone methyltransferase. Plant Cell 18, 1166–1176. 10.1105/tpc.106.041400, PMID: 16582009 PMC1456864

[ref30] El-Sharkawy I. Liang D. Xu K. N. (2015). Transcriptome analysis of an apple (*Malus domestica*) yellow fruit somatic mutation identifies a gene network module highly associated with anthocyanin and epigenetic regulation. J. Exp. Bot. 66, 7359–7376. 10.1093/jxb/erv433, PMID: 26417021 PMC4765799

[ref31] Erhard K. F. Talbot J. E. Deans N. C. McClish A. E. Hollick J. B. (2015). Nascent transcription affected by RNA polymerase IV in *Zea mays*. Genetics 199, 1107–1125. 10.1534/genetics.115.174714, PMID: 25653306 PMC4391576

[ref32] Espinas N. A. Hidetoshi S. Yusuke S. (2016). Epigenetic control of defense signaling and priming in plants. Front. Plant Sci. 7:1201. 10.3389/fpls.2016.01201, PMID: 27563304 PMC4980392

[ref33] Fedoreyeva L. I. Vanyushin B. F. (2002). N^6^-adenine DNA-methyltransferase in wheat seedlings. FEBS Lett. 514, 305–308. 10.1016/S0014-5793(02)02384-0, PMID: 11943171

[ref34] Feng S. Cokus S. J. Schubert V. Zhai J. Pellegrini M. Jacobsen S. E. (2014). Genome-wide Hi-C analyses in wild-type and mutants reveal high-resolution chromatin interactions in *Arabidopsis*. Mol. Cell 55, 694–707. 10.1016/j.molcel.2014.07.008, PMID: 25132175 PMC4347903

[ref35] Fu Y. Luo G. Z. Chen K. Deng X. Yu M.. (2015). N^6^-methyldeoxyadenosine marks active transcription start sites in *Chlamydomonas*. Cell 161, 879–892. 10.1016/j.cell.2015.04.010, PMID: 25936837 PMC4427561

[ref36] Gallego-Bartolomé J. Gardiner J. Liu W. Papikian A. Ghoshal B. Kuo H. Y.. (2018). Targeted DNA demethylation of the *Arabidopsis* genome using the human TET1 catalytic domain. Proc. Natl. Acad. Sci. U. S. A. 115, E2125–E2134. 10.1073/pnas.1716945115, PMID: 29444862 PMC5834696

[ref38] Gao Z. Liu H. L. Daxinger L. Pontes O. He X. Qian W.. (2010). An RNA polymerase II-and AGO4-associated protein acts in RNA-directed DNA methylation. Nature 465, 106–109. 10.1038/nature09025, PMID: 20410883 PMC2865564

[ref39] Gehring M. (2019). Epigenetic dynamics during flowering plant reproduction: evidence for reprogramming? New Phytol. 224, 91–96. 10.1111/nph.15856, PMID: 31002174 PMC6711810

[ref40] Gehring M. Bubb K. L. Henikoff S. (2009). Extensive demethylation of repetitive elements during seed development underlies gene imprinting. Science 324, 1447–1451. 10.1126/science.1171609, PMID: 19520961 PMC2886585

[ref41] Gehring M. Huh J. H. Hsieh T. F. Penterman J. Choi Y.. (2006). DEMETER DNA glycosylase establishes MEDEA polycomb gene self-imprinting by allele-specific demethylation. Cell 124, 495–506. 10.1016/j.cell.2005.12.034, PMID: 16469697 PMC4106368

[ref42] Gokhale N. S. McIntyre A. B. R. McFadden M. J. Roder A. E. Kennedy E. M. Gandara J. A.. (2016). N^6^-methyladenosine in flaviviridae viral RNA genomes regulates infection. Cell Host Microbe 20, 654–665. 10.1016/j.chom.2016.09.015, PMID: 27773535 PMC5123813

[ref43] Greer E. L. Blanco M. A. Gu L. Sendinc E. Liu J. Aristizabal-Corrales D.. (2015). DNA methylation on N^6^-adenine in *C. elegans*. Cell 161, 868–878. 10.1016/j.cell.2015.04.005, PMID: 25936839 PMC4427530

[ref44] Grob S. Schmid M. W. Grossniklaus U. (2014). Hi-C analysis in *Arabidopsis* identifies the KNOT, a structure with similarities to the flamenco locus of *Drosophila*. Mol. Cell 55, 678–693. 10.1016/j.molcel.2014.07.009, PMID: 25132176

[ref45] Grover J. W. Kendall T. Baten A. Burgess D. Freeling M. King G. J.. (2018). Maternal components of RNA-directed DNA methylation are required for seed development in *Brassica rapa*. Plant J. 94, 575–582. 10.1111/tpj.13910, PMID: 29569777

[ref46] He X. J. Hsu Y. F. Zhu S. Wierzbicki A. T. Pontes O. Pikaard C. S.. (2009). An effector of RNA-directed DNA methylation in *Arabidopsis* is an ARGONAUTE 4 and RNA-binding protein. Cell 137, 498–508. 10.1016/j.cell.2009.04.028, PMID: 19410546 PMC2700824

[ref47] He Y. H. Li Z. C. (2018). Epigenetic environmental memories in plants: establishment, maintenance, and reprogramming. Trends Genet. 34, 856–866. 10.1016/j.tig.2018.07.006, PMID: 30144941

[ref48] Hilker M. Schmülling T. (2019). Stress priming, memory, and signalling in plants. Plant Cell Environ. 42, 753–761. 10.1111/pce.13526, PMID: 30779228

[ref49] Hoelzer K. Shackelton L. A. Parrish C. R. (2008). Presence and role of cytosine methylation in DNA viruses of animals. Nucleic Acids Res. 36, 2825–2837. 10.1093/nar/gkn121, PMID: 18367473 PMC2396429

[ref50] Hossain M. S. Kawakatsu T. Kim K. D. Zhang N. Nguyen C. T. Khan S. M.. (2017). Divergent cytosine DNA methylation patterns in single-cell, soybean root hairs. New Phytol. 214, 808–819. 10.1111/nph.14421, PMID: 28106918

[ref51] Hsieh T. F. Ibarra C. A. Silva P. Zemach A. Eshed-Williams L.. (2009). Genome-wide demethylation of *Arabidopsis* endosperm. Science 324, 1451–1454. 10.1126/science.1172417, PMID: 19520962 PMC4044190

[ref52] Hsieh T. F. Shin J. Uzawa R. Silva P. Cohen S. Bauer M. J.. (2011). Regulation of imprinted gene expression in *Arabidopsis* endosperm. Proc. Natl. Acad. Sci. U. S. A. 108, 1755–1762. 10.1073/pnas.1019273108, PMID: 21257907 PMC3033266

[ref53] Huang W. Xiong J. Yang Y. Liu S. M. Yuan B. F. Feng Y. Q. (2015). Determination of DNA adenine methylation in genomes of mammals and plants by liquid chromatography/mass spectrometry. RSC Adv. 5, 64046–64054. 10.1039/C5RA05307B

[ref54] Huh J. H. Bauer M. J. Hsieh T. F. Fischer R. L. (2008). Cellular programming of plant gene imprinting. Cell 132, 735–744. 10.1016/j.cell.2008.02.018, PMID: 18329361

[ref55] Ibarra C. A. Feng X. Schoft V. K. Hsieh T. F. Uzawa R. Rodrigues J. A.. (2012). Active DNA demethylation in plant companion cells reinforces transposon methylation in gametes. Science 337, 1360–1364. 10.1126/science.1224839, PMID: 22984074 PMC4034762

[ref56] Ito H. Gaubert H. Bucher E. Mirouze M. Vaillant I. Paszkowski J. (2011). An siRNA pathway prevents transgenerational retrotransposition in plants subjected to stress. Nature 472, 115–119. 10.1038/nature09861, PMID: 21399627

[ref57] Iwasaki M. Paszkowski J. (2014). Identification of genes preventing transgenerational transmission of stress-induced epigenetic states. Proc. Natl. Acad. Sci. U. S. A. 111, 8547–8552. 10.1073/pnas.1402275111, PMID: 24912148 PMC4060648

[ref58] Iyer L. M. Zhang D. Aravind D. (2016). Adenine methylation in eukaryotes: apprehending the complex evolutionary history and functional potential of an epigenetic modification. BioEssays 38, 27–40. 10.1002/bies.201500104, PMID: 26660621 PMC4738411

[ref59] Johnson L. M. Du J. Hale C. J. Bischof S. Feng S. Chodavarapu R. K.. (2014). SRA-and SET-domain-containing proteins link RNA polymerase V occupancy to DNA methylation. Nature 507, 124–128. 10.1038/nature12931, PMID: 24463519 PMC3963826

[ref60] Jones M. J. Goodman S. J. Kobor M. S. (2015). DNA methylation and healthy human aging. Aging Cell 14, 924–932. 10.1111/acel.12349, PMID: 25913071 PMC4693469

[ref61] Jullien P. E. Katz A. Oliva M. Ohad N. Berger F. (2006). Polycomb group complexes self-regulate imprinting of the Polycomb group gene MEDEA in *Arabidopsis*. Curr. Biol. 16, 486–492. 10.1016/j.cub.2006.01.020, PMID: 16527743

[ref62] Jullien P. E. Mosquna A. Ingouff M. Sakata T. Ohad N. Berger F. (2008). Retinoblastoma and its binding partner MSI1 control imprinting in *Arabidopsis*. PLoS Biol. 6:e194. 10.1371/journal.pbio.0060194, PMID: 18700816 PMC2504488

[ref63] Kawashima T. Berger F. (2014). Epigenetic reprogramming in plant sexual reproduction. Nat. Rev. Genet. 15, 613–624. 10.1038/nrg3685, PMID: 25048170

[ref64] Kim J. M. To T. K. Ishida J. Matsui A. Kimura H. Seki M. (2012). Transition of chromatin status during the process of recovery from drought stress in *Arabidopsis thaliana*. Plant Cell Physiol. 53, 847–856. 10.1093/pcp/pcs053, PMID: 22505693

[ref65] Klosinska M. Picard C. L. Gehring M. (2016). Conserved imprinting associated with unique epigenetic signatures in the *Arabidopsis* genus. Nat. Plants 2, 1–8. 10.1038/nplants.2016.145, PMID: 27643534 PMC5367468

[ref66] Koltunow A. M. Grossniklaus U. (2003). Apomixis: a developmental perspective. Annu. Rev. Plant Biol. 54, 547–574. 10.1146/annurev.arplant.54.110901.160842, PMID: 14503003

[ref67] Kovacevic N. Polidoros A. N. Iliopoulos I. Tsaftaris A. S. (2005). The use of restriction landmark genome scanning (RLGS) for assessment of *Not*I-site methylation in maize. Maydica 50, 81–88.

[ref68] Kumar S. (2017). Epigenetic control of apomixis: a new perspective of an old enigma. Adv. Plants Agric. Res. 7, 10–15406. 10.15406/apar.2017.07.00243

[ref69] Kumar S. (2018). Epigenetic memory of stress responses in plants. J. Phytochem. Biochem. 2:e102. 10.3390/epigenomes2010006

[ref70] Kumar S. (2019a). Genome editing to epigenome editing: towards unravelling the enigmas in developmental biology. Trends Dev. Biol. 12, 31–38. 10.31300/TDB.12.2019.31-38

[ref71] Kumar S. (2019b). Epigenetics and epigenomics for crop improvement: current opinion. Adv. Biotechnol. Microbiol. 14:555879. 10.19080/AIBM.2019.14.555879

[ref72] Kumar S. Beena A. S. Awana M. Singh A. (2017a). Salt-induced tissue-specific cytosine methylation downregulates expression of *HKT* genes in contrasting wheat (*Triticum aestivum* L.) genotypes. DNA Cell Biol. 8, 283–294. 10.3389/fpls.2017.01151 PMC5385449 28384069

[ref73] Kumar S. Chinnusamy V. Mohapatra T. (2018). Epigenetics of modified DNA bases: 5-methylcytosine and beyond. Front. Genet. 9:640. 10.3389/fgene.2018.00640, PMID: 30619465 PMC6305559

[ref74] Kumar S. Mohapatra T. (2021). Deciphering epitranscriptome: modification of mRNA bases provides a new perspective for post-transcriptional regulation of gene expression. Front. Cell Dev. Biol. 9:628415. 10.3389/fcell.2021.628415, PMID: 33816473 PMC8010680

[ref75] Kumar S. Singh A. K. Mohapatra T. (2017b). Epigenetics: history, present status and future perspective. Indian J. Genet. Plant Breeding 77, 445–463. 10.5958/0975-6906.2017.00061.X

[ref76] La H. Ding B. Mishra G. P. Zhou B. Yang H. Bellizzi M. R.. (2011). A 5-methylcytosine DNA glycosylase/lyase demethylates the retrotransposon Tos17 and promotes its transposition in rice. Proc. Natl. Acad. Sci. U. S. A. 108, 15498–15503. 10.1073/pnas.1112704108, PMID: 21896764 PMC3174586

[ref77] Lämke J. Baeurle I. (2017). Epigenetic and chromatin-based mechanisms in environmental stress adaptation and stress memory in plants. Genome Biol. 18, 1–11. 10.1186/s13059-017-1263-6, PMID: 28655328 PMC5488299

[ref78] Lang Z. Wang Y. Tang K. Tang D. Datsenka T. Cheng J.. (2017). Critical roles of DNA demethylation in the activation of ripening-induced genes and inhibition of ripening-repressed genes in tomato fruit. Proc. Natl. Acad. Sci. U. S. A. 114, E4511–E4519. 10.1073/pnas.1705233114, PMID: 28507144 PMC5465898

[ref79] Lange U. C. Schneider R. (2010). What an epigenome remembers. BioEssays 32, 659–668. 10.1002/bies.201000030, PMID: 20658704

[ref80] Law J. A. Ausin I. Johnson L. M. Vashisht A. A. Zhu J.-K. Wohlschlegel J. A.. (2010). A protein complex required for polymerase V transcripts and RNA-directed DNA methylation in *Arabidopsis*. Curr. Biol. 20, 951–956. 10.1016/j.cub.2010.03.062, PMID: 20409711 PMC2972704

[ref81] Law J. A. Du J. Hale C. J. Feng S. Krajewski K. Palanca A. M. S.. (2013). Polymerase IV occupancy at RNA-directed DNA methylation sites requires SHH1. Nature 498, 385–389. 10.1038/nature12178, PMID: 23636332 PMC4119789

[ref82] Law J. A. Jacobsen S. E. (2010). Establishing, maintaining and modifying DNA methylation patterns in plants and animals. Nat. Rev. Genet. 11, 204–220. 10.1038/nrg2719, PMID: 20142834 PMC3034103

[ref83] Le T. N. Schumann U. Smith N. A. Tiwari S. Au P. C. Zhu Q. H.. (2014). DNA demethylases target promoter transposable elements to positively regulate stress responsive genes in *Arabidopsis*. Genome Biol. 15, 1–18. 10.1186/s13059-014-0458-3, PMID: 25228471 PMC4189188

[ref84] Lee J. Jang H. Shin H. Choi W. L. Mok Y. G. Huh J. H. (2014). AP endonucleases process 5-methylcytosine excision intermediates during active DNA demethylation in *Arabidopsis*. Nucleic Acids Res. 42, 11408–11418. 10.1093/nar/gku834, PMID: 25228464 PMC4191409

[ref85] Lei M. G. Zhang H. Julian R. Tang K. Xie S. Zhu J.-K. (2015). Regulatory link between DNA methylation and active demethylation in *Arabidopsis*. Proc. Natl. Acad. Sci. U. S. A. 112, 3553–3557. 10.1073/pnas.1502279112, PMID: 25733903 PMC4371987

[ref86] Li Q. Zynda G. Song J. Makarevitch I. Hirsch C. D. Hirsch C. N.. (2015). RNA-directed DNA methylation enforces boundaries between heterochromatin and euchromatin in the maize genome. Proc. Natl. Acad. Sci. U. S. A. 112, 14728–14733. 10.1073/pnas.1514680112, PMID: 26553984 PMC4664327

[ref87] Li Y. Córdoba-Cañero D. Qian W. Zhu X. Tang K. Zhang H.. (2015). An AP endonuclease functions in active DNA demethylation and gene imprinting in *Arabidopsis*. PLoS Genet. 11:e1004905. 10.1371/journal.pgen.1004905, PMID: 25569774 PMC4287435

[ref88] Li Y. Kumar S. Qian W. (2018a). Active DNA demethylation: mechanism and role in plant development. Plant Cell Rep. 37, 77–85. 10.1007/s00299-017-2215-z, PMID: 29026973 PMC5758694

[ref89] Li Z. Zhao P. Xia Q. (2019). Epigenetic methylations on N^6^-adenine and N^6^-adenosine with the same input but different output. Int. J. Mol. Sci. 20:2931. 10.3390/ijms20246252, PMID: 31208067 PMC6627651

[ref90] Li Z.-W. Hou X.-H. Chen J.-F. Xu Y.-C. Wu Q. González J.. (2018b). Transposable elements contribute to the adaptation of *Arabidopsis thaliana*. Genome Biol. Evol. 10, 2140–2150. 10.1093/gbe/evy171, PMID: 30102348 PMC6117151

[ref91] Liang D. Wang H. Song W. Xiong X. Zhang X. Hu Z.. (2016). The decreased N(6)-methyladenine DNA modification in cancer cells. Biochem. Biophys. Res. Commun. 480, 120–125. 10.1016/j.bbrc.2016.09.136, PMID: 27693785

[ref92] Liang Z. Riaz A. Chachar S. Ding Y. Du H. Gu X. (2020). Epigenetic modifications of mRNA and DNA in plants. Mol. Plant 13, 14–30. 10.1016/j.molp.2019.12.007, PMID: 31863849

[ref93] Liang Z. Shen L. Cui X. Bao S. Geng Y. Yu G.. (2018). DNA N^6^-adenine methylation in *Arabidopsis thaliana*. Dev. Cell 45, 406–416. 10.1016/j.devcel.2018.03.012, PMID: 29656930

[ref94] Lin J. Y. Le B. H. Chen M. Henry K. F. Hur J. Hsieh T. F.. (2017). Similarity between soybean and *Arabidopsis* seed methylomes and loss of non-CG methylation does not affect seed development. Proc. Natl. Acad. Sci. U. S. A. 114, E9730–E9739. 10.1073/pnas.1716758114, PMID: 29078418 PMC5692608

[ref95] Lippman Z. Gendrel A.-V. Black M.. (2004). Role of transposable elements in heterochromatin and epigenetic control. Nature 430, 471–476. 10.1038/nature02651, PMID: 15269773

[ref96] Lister R. O'Malley R. C. Tonti-Filippini J. Gregory B. D. Berry C. C. Millar A. H.. (2008). Highly integrated single-base resolution maps of the epigenome in *Arabidopsis*. Cell 133, 523–536. 10.1016/j.cell.2008.03.029, PMID: 18423832 PMC2723732

[ref97] Liu R. How-Kit A. Stammitti L. Teyssier E. Rolin D. Mortain-Bertrand A.. (2015). A DEMETER-like DNA demethylase governs tomato fruit ripening. Proc. Natl. Acad. Sci. U. S. A. 112, 10804–10809. 10.1073/pnas.1503362112, PMID: 26261318 PMC4553810

[ref98] Liu X. S. Wu H. Ji X. Stelzer Y. Wu X. Czauderna S.. (2016). Editing DNA methylation in the mammalian genome. Cell 167, 233–247. 10.1016/j.cell.2016.08.056, PMID: 27662091 PMC5062609

[ref99] Liu Z. L. Han F. P. Tan M. Shan X. H. Dong Y. Z.. (2004). Activation of a rice endogenous retrotransposon Tos17 in tissue culture is accompanied by cytosine demethylation and causes heritable alteration in methylation pattern of flanking genomic regions. Theor. Appl. Genet. 109, 200–209. 10.1007/s00122-004-1618-8, PMID: 15071728

[ref100] Liu Z.-W. Shao C.-R. Zhang C.-J. Zhou J. X. Zhang S.-W. Li L.. (2014). The SET domain proteins SUVH2 and SUVH9 are required for pol V occupancy at RNA-directed DNA methylation loci. PLoS Genet. 10:e1003948. 10.1371/journal.pgen.1003948, PMID: 24465213 PMC3898904

[ref101] Lopez Sanchez A. Stassen J. H. Furci L. Smith L. M. Ton J. (2016). The role of DNA (de)methylation in immune responsiveness of *Arabidopsis*. Plant J. 88, 361–374. 10.1111/tpj.13252, PMID: 27341062 PMC5132069

[ref104] Luo M. Taylor J. M. Spriggs A. Zhang H. Wu X.. (2011). A genome-wide survey of imprinted genes in rice seeds reveals imprinting primarily occurs in the endosperm. PLoS Genet. 7:e1002125. 10.1371/journal.pgen.1002125, PMID: 21731498 PMC3121744

[ref105] Manning K. Tor M. Poole M. Hong Y. Thompson A. J. King G. J.. (2006). A naturally occurring epigenetic mutation in a gene encoding an SBP-box transcription factor inhibits tomato fruit ripening. Nat. Genet. 38, 948–952. 10.1038/ng1841, PMID: 16832354

[ref106] Martínez G. Panda K. Köhler C. Slotkin R. K. (2016). Silencing in sperm cells is directed by RNA movement from the surrounding nurse cell. Nat. Plants. 2, 1–8. 10.1038/nplants.2016.30, PMID: 27249563

[ref107] Martinez-Macias M. I. Qian W. Miki D. Pontes O. Liu Y. Tang K.. (2012). A DNA 3′ phosphatase functions in active DNA demethylation in *Arabidopsis*. Mol. Cell 45, 357–370. 10.1016/j.molcel.2011.11.034, PMID: 22325353 PMC3278721

[ref004] Masuta Y. Nozawa K. Takagi H. Yaegashi H. Tanaka K. Ito T. J.. (2017). Inducible transposition of a heat-activated retrotransposon in tissue culture. Plant Cell Physiol. 58, 375–384. 10.1093/pcp/pcw202, PMID: 28013279

[ref108] Matzke M. A. Mosher R. A. (2014). RNA-directed DNA methylation: an epigenetic pathway of increasing complexity. Nat. Rev. Genet. 15, 394–408. 10.1038/nrg3683, PMID: 24805120

[ref109] Mayer W. Niveleau A. Walter J. Fundele R. Haaf T. (2000). Demethylation of the zygotic paternal genome. Nature 403, 501–502. 10.1038/35000656, PMID: 10676950

[ref110] McCue A. D. Panda K. Nuthikattu S. Choudury S. G. Thomas E. N. Slotkin R. K. (2015). ARGONAUTE 6 bridges transposable element mRNA-derived siRNAs to the establishment of DNA methylation. EMBO J. 34, 20–35. 10.15252/embj.201489499, PMID: 25388951 PMC4291478

[ref111] Mendizabal I. Yi S. V. (2016). Whole-genome bisulfite sequencing maps from multiple human tissues reveal novel CpG island associated with tissue-specific regulation. Hum. Mol. Genet. 25, 69–82. 10.1093/hmg/ddv449 26512062 PMC4690492

[ref112] Mirouze M. Reinders J. Bucher E. Nishimura T. Schneeberger K. Ossowski S.. (2009). Selective epigenetic control of retrotransposition in *Arabidopsis*. Nature 461, 427–430. 10.1038/nature08328, PMID: 19734882

[ref113] Morales-Ruiz T. Ortega-Galisteo A. P. Ponferrada-Marín M. I. Martínez-Macías M. I. Ariza R. R. Roldán-Arjona T. (2006). DEMETER and REPRESSOR OF SILENCING 1 encode 5-methylcytosine DNA glycosylases. Proc. Natl. Acad. Sci. U. S. A. 103, 6853–6858. 10.1073/pnas.0601109103, PMID: 16624880 PMC1458983

[ref114] Nuthikattu S. McCue A. D. Panda K. Fultz D. DeFraia C. Thomas E. N.. (2013). The initiation of epigenetic silencing of active transposable elements is triggered by RDR6 and 21–22 nucleotide small interfering RNAs. Plant Physiol. 162, 116–131. 10.1104/pp.113.216481, PMID: 23542151 PMC3641197

[ref115] Ooi L. Belyaev N. D. Miyake K. Wood I. C. Buckley N. J. (2006). BRG1 chromatin remodeling activity is required for efficient chromatin binding by repressor element 1-silencing transcription factor (REST) and facilitates REST-mediated repression. J. Biol. Chem. 281, 38974–38980. 10.1074/jbc.M605370200, PMID: 17023429 PMC1820614

[ref116] Ortega-Galisteo A. P. Morales-Ruiz T. Ariza R. R. Roldan-Arjona T. (2008). *Arabidopsis* DEMETER-LIKE proteins DML2 and DML3 are required for appropriate distribution of DNA methylation marks. Plant Mol. Biol. 67, 671–681. 10.1007/s11103-008-9346-0, PMID: 18493721

[ref001] Park K. Kim M. Y. Vickers M. Park J. S. Hyun Y. Okamoto T.. (2016). DNA demethylation is initiated in the central cells of Arabidopsis and rice. Proc. Natl. Acad. Sci. U. S. A. 113, 15138–15143. 10.1073/pnas.1619047114 27956642 PMC5206524

[ref119] Pecinka A. Chevalier C. Colas I. Kalantidis K. Varotto S. Krugman T.. (2019). Chromatin dynamics during interphase and cell division: similarities and differences between model and crop plants. J. Exp. Bot. 71, 5205–5222. 10.1093/jxb/erz457 31626285

[ref120] Penterman J. Zilberman D. Huh J. H. Ballinger T. Henikoff S. Fischer R. L. (2007). DNA demethylation in the *Arabidopsis* genome. Proc. Natl. Acad. Sci. U. S. A. 104, 6752–6757. 10.1073/pnas.0701861104, PMID: 17409185 PMC1847597

[ref121] Pukkila P. J. Peterson J. Herman G. Modrich P. Meselson M. (1983). Effects of high levels of DNA adenine methylation on methyl-directed mismatch repair in *Escherichia coli*. Genetics 104, 571–582. 10.1093/genetics/104.4.571, PMID: 6225697 PMC1202127

[ref122] Pumplin N. Voinnet O. (2013). RNA silencing suppression by plant pathogens: defence, counter-defence and counter-counter-defence. Nat. Rev. Microbiol. 11, 745–760. 10.1038/nrmicro3120, PMID: 24129510

[ref123] Qian W. Miki D. Zhang H. Liu Y. Zhang X. Tang K.. (2012). A histone acetyltransferase regulates active DNA demethylation in *Arabidopsis*. Science 336, 1445–1448. 10.1126/science.1219416, PMID: 22700931 PMC3575687

[ref124] Raja P. Wolf J. N. Bisaro D. M. (2010). RNA silencing directed against geminiviruses: post-transcriptional and epigenetic components. Biochim. Biophys. Acta 1799, 337–351. 10.1016/j.bbagrm.2010.01.004, PMID: 20079472

[ref125] Ratel D. Ravanat J. L. Berger F. Wion D. (2006). N^6^-methyladenine: the other methylated base of DNA. BioEssays 28, 309–315. 10.1002/bies.20342, PMID: 16479578 PMC2754416

[ref126] Rathore P. Raina S. N. Kumar S. Bhat V. (2020). Retro-element Gypsy-163 is differentially methylated in reproductive tissues of apomictic and sexual plants of *Cenchrus ciliaris*. Front. Genet. 11:795. 10.3389/fgene.2020.00795, PMID: 32849800 PMC7387646

[ref127] Rodrigues J. A. Ruan R. Nishimura T. Sharma M. K. Sharma R.. (2013). Imprinted expression of genes and small RNA is associated with localized hypomethylation of the maternal genome in rice endosperm. Proc. Natl. Acad. Sci. U. S. A. 110, 7934–7939. 10.1073/pnas.1306164110, PMID: 23613580 PMC3651473

[ref128] Rodríguez-Negrete E. A. Carrillo-Tripp J. Rivera-Bustamante R. F. (2009). RNA silencing against geminivirus: complementary action of posttranscriptional gene silencing and transcriptional gene silencing in host recovery. J. Virol. 83, 1332–1340. 10.1128/JVI.01474-08, PMID: 19019951 PMC2620903

[ref129] Rowley M. J. Rothi M. H. Bohmdorfer G. Kucinski J. Wierzbicki A. T. (2017). Long-range control of gene expression *via* RNA-directed DNA methylation. PLoS Genet. 13:e1006749. 10.1371/journal.pgen.1006749, PMID: 28475589 PMC5438180

[ref130] Sahu P. P. Pandey G. Sharma N. Puranik S. Muthamilarasan M. Prasad M. (2013). Epigenetic mechanisms of plant stress responses and adaptation. Plant Cell Rep. 32, 1151–1159. 10.1007/s00299-013-1462-x, PMID: 23719757

[ref131] Sanchez D. H. Paszkowski J. (2014). Heat-induced release of epigenetic silencing reveals the concealed role of an imprinted plant gene. PLoS Genet. 10:e1004806. 10.1371/journal.pgen.1004806, PMID: 25411840 PMC4238952

[ref132] Secco D. Wang C. Shou H. Schultz M. D. Chiarenza S. Nussaume L.. (2015). Stress induced gene expression drives transient DNA methylation changes at adjacent repetitive elements. eLife 4:e09343. 10.7554/eLife.09343, PMID: 26196146 PMC4534844

[ref133] Sedgwick B. Bates P. A. Paik J. Jacobs S. C. Lindahl T. (2007). Repair of alkylated DNA: recent advances. DNA Repair 6, 429–442. 10.1016/j.dnarep.2006.10.005, PMID: 17112791

[ref134] Seymour D. K. Koenig D. Hagmann J. Becker C. Weigel D. (2014). Evolution of DNA methylation patterns in the *Brassicaceae* is driven by differences in genome organization. PLoS Genet. 10:e1004785. 10.1371/journal.pgen.1004785, PMID: 25393550 PMC4230842

[ref135] Shah K. Cao W. Ellison C. E. (2019). Adenine methylation in *Drosophila* is associated with the tissue-specific expression of developmental and regulatory genes. Genes Genomes Genet. 9, 1893–1900. 10.1534/g3.119.400023 PMC6553526 30988038

[ref136] Sharma N. Sahu P. P. Puranik S. Prasad M. (2013). Recent advances in plant–virus interaction with emphasis on small interfering RNAs (siRNAs). Mol. Biotechnol. 55, 63–77. 10.1007/s12033-012-9615-7, PMID: 23086491

[ref137] She W. Baroux C. (2015). Chromatin dynamics in pollen mother cells underpin a common scenario at the somatic-to-reproductive fate transition of both the male and female lineages in *Arabidopsis*. Front. Plant Sci. 6:294. 10.3389/fpls.2015.00294, PMID: 25972887 PMC4411972

[ref138] Slaughter A. Daniel X. Flors V. Luna E. Hohn B. Mauch-Mani B. (2012). Descendants of primed *Arabidopsis* plants exhibit resistance to biotic stress. Plant Physiol. 158, 835–843. 10.1104/pp.111.191593, PMID: 22209872 PMC3271771

[ref139] Slotkin R. K. Martienssen R. (2007). Transposable elements and the epigenetic regulation of the genome. Nat. Rev. Genet. 8, 272–285. 10.1038/nrg2072, PMID: 17363976

[ref140] Stroud H. Do T. Du J. Zhong X. Feng S. Johnson L.. (2014). Non-CG methylation patterns shape the epigenetic landscape in *Arabidopsis*. Nat. Struct. Mol. Biol. 21, 64–72. 10.1038/nsmb.2735, PMID: 24336224 PMC4103798

[ref141] Stroud H. Greenberg M. V. C. Feng S. H. Bernatavichute Y. V. Jacobsen S. E. (2013). Comprehensive analysis of silencing mutants reveals complex regulation of the *Arabidopsis* methylome. Cell 152, 352–364. 10.1016/j.cell.2012.10.054, PMID: 23313553 PMC3597350

[ref142] Stuart T. Eichten S. R. Cahn J. Karpievitch Y. V. Borevitz J. O. Lister R. (2016). Population scale mapping of transposable element diversity reveals links to gene regulation and epigenomic variation. eLife 5:e20777. 10.7554/eLife.20777, PMID: 27911260 PMC5167521

[ref143] Taghbalout A. Du M. Jillette N. Rosikiewicz W. Rath A. Heinen C. D.. (2016). Enhanced CRISPR-based DNA demethylation by Casilio-ME-mediated RNA-guided coupling of methylcytosine oxidation and DNA repair pathways. Nat. Commun. 10, 1–12. 10.1038/s41467-019-12339-7, PMID: 31541098 PMC6754513

[ref144] Takuno S. Gaut B. S. (2013). Gene body methylation is conserved between plant orthologs and is of evolutionary consequence. Proc. Natl. Acad. Sci. U. S. A. 110, 1797–1802. 10.1073/pnas.1215380110, PMID: 23319627 PMC3562806

[ref145] Tan F. Zhou C. Zhou Q. Zhou S. Yang W. Zhao Y.. (2016). Analysis of chromatin regulators reveals specific features of rice DNA methylation pathways. Plant Physiol. 171, 2041–2054. 10.1104/pp.16.00393, PMID: 27208249 PMC4936571

[ref146] Tang K. Lang Z. Zhang H. Zhu J.-K. (2016). The DNA demethylase ROS1 targets genomic regions with distinct chromatin modifications. Nat. Plants 2:16169. 10.1038/nplants.2016.169, PMID: 27797352 PMC5123759

[ref147] Telias A. Wang K. Stevenson D. Coone J. Hellens R. Allan A.. (2011). Apple skin patterning is associated with differential expression of MYB10. BMC Plant Biol. 11, 1–15. 10.1186/1471-2229-11-93, PMID: 21599973 PMC3127826

[ref148] Thiebaut F. Hemerly A. S. Ferreira P. C. G. (2019). A role for epigenetic regulation in the adaptation and stress responses of non-model plants. Front. Plant Sci. 10:246. 10.3389/fpls.2019.00246, PMID: 30881369 PMC6405435

[ref149] Tricker P. J. Gibbings J. G. Rodriguez Lopez C. M. Hadley P. Wilkinson M. J. (2012). Low relative humidity triggers RNA-directed de novo DNA methylation and suppression of genes controlling stomatal development. J. Exp. Bot. 63, 3799–3813. 10.1093/jxb/ers076, PMID: 22442411 PMC3733579

[ref150] Tsukahara S. Kobayashi A. Kawabe A. Mathieu O. Miura A. Kakutani T. (2009). Bursts of retrotransposition reproduced in *Arabidopsis*. Nature 461, 423–426. 10.1038/nature08351, PMID: 19734880

[ref151] Vanyushin B. F. (2005). Adenine methylation in eukaryotic DNA. Mol. Biol. 39, 473–481. 10.1007/s11008-005-0064-2

[ref152] Varotto S. Tani E. Abraham E. Krugman T. Kapazoglou A. Melzer R.. (2020). Epigenetics: possible applications in climate-smart crop breeding. J. Exp. Bot. 71, 5223–5236. 10.1093/jxb/eraa188, PMID: 32279074 PMC7475248

[ref153] Verhoeven K. J. Jansen J. J. van Dijk P. J. Biere A. (2010). Stress-induced DNA methylation changes and their heritability in asexual dandelions. New Phytol. 185, 1108–1118. 10.1111/j.1469-8137.2009.03121.x, PMID: 20003072

[ref154] Vu T. M. Nakamura M. Calarco J. P. Susaki D. Lim P. Q. Kinoshita T.. (2013). RNA-directed DNA methylation regulates parental genomic imprinting at several loci in *Arabidopsis*. Development 140, 2953–2960. 10.1242/dev.092981, PMID: 23760956 PMC3879202

[ref155] Walker J. Gao H. Zhang J. Aldridge B. Vickers M. Higgins J. D.. (2018). Sexual-lineage-specific DNA methylation regulates meiosis in *Arabidopsis*. Nat. Genet. 50, 130–137. 10.1038/s41588-017-0008-5, PMID: 29255257 PMC7611288

[ref156] Wang B. Yang X. Wang Y. Xie Y. Zhou X. (2018a). Tomato yellow leaf curl virus V2 interacts with host histone deacetylase 6 to suppress methylation-mediated transcriptional gene silencing in plants. J. Virol. 92:e00036-18. 10.1128/jvi.00036-18, PMID: 29950418 PMC6146709

[ref157] Wang C. Wang C. Xu W. Zou J. Qiu Y. Kong J.. (2018b). Epigenetic changes in the regulation of *Nicotiana tabacum* response to cucumber mosaic virus infection and symptom recovery through single-base resolution methylomes. Viruses 10:402. 10.3390/v10080402, PMID: 30060626 PMC6115852

[ref158] Wang C. Wang C. Zou J. Yang Y. Li Z. Zhu S. (2019). Epigenetics in the plant–virus interaction. Plant Cell Rep. 38, 1031–1038. 10.1007/s00299-019-02414-0, PMID: 31065780

[ref159] Wang M.-B. Masuta C. Smith N. A. Shimura H. (2012). RNA silencing and plant viral diseases. Mol. Plant-Microbe Interact. 25, 1275–1285. 10.1094/MPMI-04-12-0093-CR, PMID: 22670757

[ref160] Wang X. Li Q. Yuan W. Cao Z. Qi B. Kumar S.. (2016). The cytosolic Fe–S cluster assembly component MET18 is required for the full enzymatic activity of ROS1 in active DNA demethylation. Sci. Rep. 6, 1–15. 10.1038/srep39807, PMID: 27193999 PMC4872223

[ref161] Waters A. J. Makarevitch I. Eichten S. R. Swanson-Wagner R. A. Yeh C. T.. (2011). Parent-of-origin effects on gene expression and DNA methylation in the maize endosperm. Plant Cell 23, 4221–4233. 10.1105/tpc.111.092668, PMID: 22198147 PMC3269861

[ref162] Wei L. Cao X. (2016). The effect of transposable elements on phenotypic variation: insights from plants to humans. Sci. China Life Sci. 59, 24–37. 10.1007/s11427-015-4993-2 26753674

[ref163] Wei L. Gu L. Song X. Cui X. Lu Z. Zhou M.. (2014). Dicer-like 3 produces transposable element-associated 24-nt siRNAs that control agricultural traits in rice. Proc. Natl. Acad. Sci. U. S. A. 111, 3877–3882. 10.1073/pnas.1318131111, PMID: 24554078 PMC3956178

[ref164] Wei W. Tao J. J. Chen H. W. Li Q. T. Zhang W. K. Ma B.. (2017). A histone code reader and a transcriptional activator interact to regulate genes for salt tolerance. Plant Physiol. 175, 1304–1320. 10.1104/pp.16.01764, PMID: 28874519 PMC5664453

[ref003] Westbye M. P. Feyzl E. Aas P. A. Vagbø C. B. Talstad V. A. Kavll B.. (2008). Human AlkB homolog I is a mitochondrial protein that demethylates 3-methylcytosine in DNA and RNA. J. Biol. Chem. 283, 25046–25056. 10.1074/jbc.M803776200, PMID: 18603530 PMC3259822

[ref165] Wibowo A. Becker C. Marconi G. Durr J. Price J. Hagmann J.. (2016). Hyperosmotic stress memory in *Arabidopsis* is mediated by distinct epigenetically labile sites in the genome and is restricted in the male germline by DNA glycosylase activity. eLife 5:e13546. 10.7554/eLife.13546, PMID: 27242129 PMC4887212

[ref166] Wierzbicki A. T. Haag J. R. Pikaard C. S. (2008). Noncoding transcription by RNA polymerase pol IVb/pol V mediates transcriptional silencing of overlapping and adjacent genes. Cell 135, 635–648. 10.1016/j.cell.2008.09.035, PMID: 19013275 PMC2602798

[ref167] Williams B. P. Pignatta D. Henikoff S. Gehring M. (2015). Methylation-sensitive expression of a DNA demethylase gene serves as an epigenetic rheostat. PLoS Genet. 11:e1005142. 10.1371/journal.pgen.1005142, PMID: 25826366 PMC4380477

[ref168] Woo H. R. Dittmer T. A. Richards E. J. (2008). Three SRA domain methylcytosine-binding proteins cooperate to maintain global CpG methylation and epigenetic silencing in *Arabidopsis*. PLoS Genet. 4:e1000156. 10.1371/journal.pgen.1000156, PMID: 18704160 PMC2491724

[ref169] Wu L. Mao L. Qi Y. (2012). Roles of dicer-like and argonaute proteins in TAS-derived small interfering RNA-triggered DNA methylation. Plant Physiol. 160, 990–999. 10.1104/pp.112.200279, PMID: 22846193 PMC3461571

[ref170] Wu X. Zhang Y. (2017). TET-mediated active DNA demethylation: mechanism, function and beyond. Nat. Rev. Genet. 18, 517–534. 10.1038/nrg.2017.33, PMID: 28555658

[ref171] Xiang H. Zhu J. Chen Q. Dai F. Li X. Li M.. (2010). Single base-resolution methylome of the silkworm reveals a sparse epigenomic map. Nat. Biotechnol. 28, 516–520. 10.1038/nbt.1626, PMID: 20436463

[ref172] Xiao C. L. Zhu S. He M. Chen Z. Q. Chen Y.. (2018). N^6^-Methyladenine DNA modification in the human genome. Mol. Cell 71, 306–318.e7. 10.1016/j.molcel.2018.06.015, PMID: 30017583

[ref173] Xing C. Liu Y. Zhao L. Zhang S. Huang X. (2018). A novel MYB transcription factor regulates ascorbic acid synthesis and affects cold tolerance. Plant Cell Environ. 42, 832–845. 10.1111/pce.13387 29929211

[ref174] Xing M.-Q. Zhang Y.-J. Zhou S.-R. Hu W.-Y. Wu X. T. Ye Y.-J.. (2015). Global analysis reveals the crucial roles of DNA methylation during rice seed development. Plant Physiol. 168, 1417–1432. 10.1104/pp.15.00414, PMID: 26145151 PMC4528747

[ref175] Yang D. L. Zhang G. Tang K. Li J. Yang L. Huang H.. (2016). Dicer-independent RNA-directed DNA methylation in *Arabidopsis*. Cell Res. 26, 66–82. 10.1038/cr.2015.145, PMID: 26642813 PMC4816133

[ref176] Yao B. Li Y. Wang Z. Chen L. Poidevin M. Zhang C.. (2018). Active N^6^-methyladenine demethylation by DMAD regulates gene expression by coordinating with polycomb protein in neurons. Mol. Cell 71, 848–857. 10.1016/j.molcel.2018.07.005, PMID: 30078725 PMC6136845

[ref177] Ye R. Q.. (2016). A dicer-independent route for biogenesis of siRNAs that direct DNA methylation in *Arabidopsis*. Mol. Cell 61, 222–235. 10.1016/j.molcel.2015.11.015, PMID: 26711010 PMC5110219

[ref178] Zemach A. Kim M. Y. Hsieh P. H. Coleman-Derr D. Eshed-Williams L. Thao K.. (2013). The *Arabidopsis* nucleosome remodeler DDM1 allows DNA methyltransferases to access H1-containing heterochromatin. Cell 153, 193–205. 10.1016/j.cell.2013.02.033, PMID: 23540698 PMC4035305

[ref179] Zhang G. Huang H. Liu D. Cheng Y. Liu X. Zhang W.. (2015). N(6)-methyladenine DNA modification in *Drosophila*. Cell 161, 893–906. 10.1016/j.cell.2015.04.018, PMID: 25936838

[ref180] Zhang H. He X. Zhu J.-K. (2013a). DTF1 is a core component of RNA-directed DNA methylation and may assist in the recruitment of pol IV. Proc. Natl. Acad. Sci. U. S. A. 110, 8290–8295. 10.1073/pnas.1300585110, PMID: 23637343 PMC3657815

[ref181] Zhang H. Lang Z. Zhu J.-K. (2018a). Dynamics and function of DNA methylation in plants. Nat. Rev. Mol. Cell Biol. 19, 489–506. 10.1038/s41580-018-0016-z 29784956

[ref182] Zhang M. Xie S. Dong X. Zhao X. Zeng B. Chen J.. (2014). Genome-wide high resolution parental-specific DNA and histone methylation maps uncover patterns of imprinting regulation in maize. Genome Res. 24, 167–176. 10.1101/gr.155879.113, PMID: 24131563 PMC3875858

[ref183] Zhang M. Xu C. von Wettstein D. Liu B. (2011). Tissue-specific differences in cytosine methylation and their association with differential gene expression in *Sorghum bicolar*. Plant Physiol. 156, 1955–1966. 10.1104/pp.111.176842, PMID: 21632971 PMC3149958

[ref184] Zhang Q. Li Y. Xu T.. (2016). The chromatin remodeler DDM1 promotes hybrid vigor by regulating salicylic acid metabolism. Cell Discov. 2, 1–12. 10.1038/celldisc.2016.27, PMID: 27551435 PMC4977722

[ref185] Zhang Q. Liang Z. Cui X. Ji C. Li Y. Zhang P.. (2018b). N^6^-methyladenine DNA methylation in japonica and Indica rice genomes and its association with gene expression, plant development, and stress responses. Mol. Plant 11, 1492–1508. 10.1016/j.molp.2018.11.005, PMID: 30448535

[ref186] Zhang X. Yazaki J. Sundaresan A. Cokus S. Chan S. W.-L. Chen H.. (2006). Genome-wide high-resolution mapping and functional analysis of DNA methylation in *Arabidopsis*. Cell 126, 1189–1201. 10.1016/j.cell.2006.08.003, PMID: 16949657

[ref187] Zhang Y.-Y. Fischer M. Colot V. V. Bossdorf O. (2013b). Epigenetic variation creates potential for evolution of plant phenotypic plasticity. New Phytol. 197, 314–322. 10.1111/nph.12010 23121242

[ref188] Zheng X. Chen L. Xia H. Wei H. Lou Q. Li M.. (2017). Transgenerational epimutations induced by multi-generation drought imposition mediate rice plant’s adaptation to drought condition. Sci. Rep. 7:17885. 10.1038/s41598-017-18273-2, PMID: 28051176 PMC5209664

[ref189] Zhong S. Fei Z. Chen Y.-R. Zheng Y. Huang M. Vrebalov J.. (2013). Single-base resolution methylomes of tomato fruit development reveal epigenome modifications associated with ripening. Nat. Biotechnol. 31, 154–159. 10.1038/nbt.2462, PMID: 23354102

[ref190] Zhong X. Du J. Hale C. J. Gallego-Bartolome J. Feng S. Vashisht A. A.. (2014). Molecular mechanism of action of plant DRM de novo DNA methyltransferases. Cell 157, 1050–1060. 10.1016/j.cell.2014.03.056, PMID: 24855943 PMC4123750

[ref191] Zhong X. Hale C. J. Law J. A. Johnson L. M. Feng S. Tu A.. (2012). DDR complex facilitates global association of RNA polymerase V to promoters and evolutionarily young transposons. Nat. Struct. Mol. Biol. 19, 870–875. 10.1038/nsmb.2354, PMID: 22864289 PMC3443314

[ref192] Zhou C. Wang C. Liu H. Zhou Q. Liu Q. Guo Y. (2018). Identification and analysis of adenine N^6^-methylation sites in the rice genome. Nat. Plant 4, 554–563. 10.1038/s41477-018-0214-x, PMID: 30061746

[ref193] Zhu J. H. Kapoor A. Sridhar V. V. Agius F. Zhu J.-K. (2007). The DNA glycosylase/lyase ROS1 functions in pruning DNA methylation patterns in *Arabidopsis*. Curr. Biol. 17, 54–59. 10.1016/j.cub.2006.10.059, PMID: 17208187

[ref194] Zhu Q. H. Shan W. X. Ayliffe M. Wang M. B. (2015). Epigenetic mechanisms: an emerging player in plant–microbe interactions. Mol. Plant-Microbe Interact. 29, 187–196. 10.1094/MPMI-08-15-0194-FI 26524162

[ref195] Zilberman D. Gehring M. Tran R. K. Ballinger T. Henikoff S. (2006). Genome-wide analysis of *Arabidopsis thaliana* DNA methylation uncovers an interdependence between methylation and transcription. Nat. Genet. 39, 61–69. 10.1038/ng1929 17128275

